# Supplementary material for: Selective Oxidation of Clopidogrel by Peroxymonosulfate (PMS) and Sodium Halide (NaX) System: An NMR Study
Source: Molecules. 2021 Sep 29;26(19):5921. doi: 10.3390/molecules26195921 (PMC8512727; doi:10.3390/molecules26195921)
Supplement: Supplementary file 1 [file molecules-26-05921-s001.zip › molecules-1368882-supplementary.pdf]

Selective Oxidation of Clopidogrel by Peroxymonosulfate (PMS) and Sodium Halide (NaX) System: An NMR Study

Everaldo F. Krake and Wolfgang Baumann *

Leibniz-Institut für Katalyse e.V., Albert-Einstein-Straße 29a, 18059 Rostock, Germany; everaldokiko@gmail.com
* Correspondence: wolfgang.baumann@catalysis.de

Supplementary Materials

Summary	Page
Analytical data	S2
HPLC-MS chromatogram: PMS/NaCl	S3
HPLC chromatograms	S4
UV spectrum	S5
HR-MS / NMR spectrum	S6

Analytical data

(S)-5-(1-(2-chlorophenyl)-2-methoxy-2-oxoethyl)-6,7-dihydrothieno[3,2-c]pyridin-5-ium (DP-2)

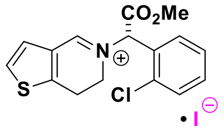

This compound is known, but the counterion is new.[1]
Yellow solid. ^{1}**H NMR** (400 MHz, CD_3CN) δ 8.81 (s, 1H), 7.62 – 7.49 (m, 6H), 6.40 (s, 1H), 4.30 – 4.23 (m, 1H), 3.95 – 3.86 (m, 1H), 3.89 (s, 3H), 3.52 – 3.36 (m, 2H); ^{13}**C NMR** (101 MHz, CD_3CN) δ 167.81, 162.40, 156.50, 136.18, 133.81, 132.86, 131.83, 129.44, 129.35, 129.16, 129.02, 128.15, 72.08, 55.02, 49.94, 24.04. **HRMS** (ESI) m/z [M+H] calcd. for $C_{16}H_{15}ClNO_2S^+$: 320,0517, found 320.0516; –0.3 ppm error.

Methyl (S)-2-(2-chloro-6,7-dihydrothieno[3,2-c]pyridin-5(4H)-yl)-2-(2-chlorophenyl)acetate (DP-3a)

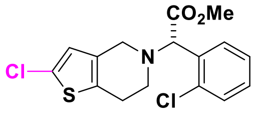

Spectra matched the literature.[2]
White solid. ^{1}**H NMR** (400 MHz, CD_3OD) δ 7.63 – 7.60 (m, 1H), 7.44 – 7.41 (m, 1H), 7.33 – 7.29 (m, 3H), 6.50 (s, 1H), 4.97 (s, 1H), 3.68 – 3.66 (m, 4H), 3.62 (d, *J* = 2.1 Hz, 1H), 3.56 – 3.52 (d, *J* = 2.1 Hz, 1H), 2.98 – 2.84 (m, 2H), 2.76 – 2.70 (m, 2H). ^{13}**C NMR** (101 MHz, CD_3OD) δ 172.10, 135.80, 134.13, 133.59, 133.28, 131.11, 130.99 (2CH), 128.48, 128.22, 125.54, 68.55, 52.84, 51.07, 49.21, 25.78. **HRMS** (ESI) m/z [M+H] calcd. for $C_{16}H_{11}O_2NSCl_2$: 356.0279 found: 356.0288; 2.5 ppm error.

2-chloro-5-((S)-1-(2-chlorophenyl)-2-methoxy-2-oxoethyl)-5-hydroxy-4,5,6,7-tetrahydrothieno[3,2-c]pyridin-5-ium (DP-4a)

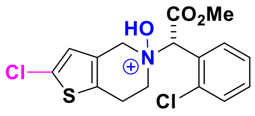

This diastereomeric product is unknown, unstable and was not isolated. ^{1}**H NMR** (400 MHz, CD_3CN) signals correspond to 56:44 mixture of diastereomers: ^{1}H NMR (400 MHz, Acetonitrile-d_3) δ .77 (dd, *J* = 7.9, 1.6 Hz, 1H), 7.73 (dd, *J* = 7.9, 1.6 Hz, 1H), 7.65 – 7.53 (m, 4H), 7.50 – 7.43 (m, 2H), 6.70 (s, 1H), 6.60 (s, 1H), 6.19 (s, 1H), 6.15 (s, 1H), 5.09 (d, *J* = 15.5 Hz, 1H), 4.82 (d, *J* = 15.5 Hz, 1H), 4.65 (s, 2H), 4.29 (dd, *J* = 9.2, 5.1 Hz, 1H), 4.22 (dd, *J* = 12.1, 5.3 Hz, 1H), 4.14 – 3.97 (m, 1H), 3.75 (s, 6H), 3.74 (s, 5H), 3.33 – 3.14 (m, 2H), 3.15 – 2.98 (m, 2H). ^{13}**C NMR** (101 MHz, CD_3CN) δ 166.27, 166.17, 137.59, 137.17, 134.58, 134.55, 131.91, 131.83, 130.56, 130.48, 130.39, 130.18, 129.20, 129.09, 125.72, 125.39, 125.26, 76.95, 74.93, 62.50, 62.27, 61.99, 60.68, 55.06, 22.19, 21.54. **HRMS** (ESI) m/z [M+H] calcd. for $C_{16}H_{15}O_3NSCl_2$: 372.0228 found: 372.0227; –0.3 ppm error.

Methyl (S)-2-(2-bromo-6,7-dihydrothieno[3,2-c]pyridin-5(4H)-yl)-2-(2-chlorophenyl)acetate (DP-3b)

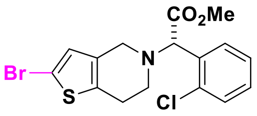

This compound is known, but the analytical data is not shown.[3]
Orange oil. ^{1}**H NMR** (400 MHz, CD_3OD) δ 7.66 – 7.61 (m, 1H), 7.48 – 7.43 (m, 1H), 7.37 – 7.31 (m, 2H), 6.70 (s, 1H), 4.93 (s, 1H), 3.70 (s, 3H), 3.63 (dt, *J* = 14.4, 1.9 Hz, 1H), 3.57 – 3.50 (m, 1H), 2.95 – 2.80 (m, 2H), 2.76 (tq, *J* = 6.4, 1.9 Hz, 2H); ^{13}**C NMR** (101 MHz, CD_3OD) δ 172.76, 136.39, 135.91, 135.32, 134.76, 131.05, 131.02 (2CH), 129.36, 128.50, 110.38, 68.68, 52.71, 51.10, 49.21, 26.02. **HRMS** (ESI) m/z [M+H] calcd. for $C_{16}H_{11}O_2NSClBr$: 399.9774 found: 399.9772; –0.5 ppm error.

(S)-2-bromo-5-(1-(2-chlorophenyl)-2-methoxy-2-oxoethyl)-6,7-dihydrothieno[3,2-c]pyridin-5-ium (DP-5b)

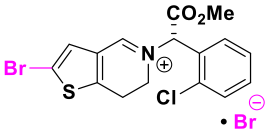

This compound is unknown.
Yellow solid. ^{1}**H NMR** (400 MHz, CD_3OD) δ 9.05 (s, 1H), 7.68 – 7.56 (m, 5H), 6.62 (s, 1H), 4.44 – 4.37 (m, 1H), 4.00 – 3.91 (m, 1H), 3.97 (s, 3H), 3.59 – 3.40 (m, 2H); ^{13}**C NMR** (101 MHz, CD_3OD) δ 168.19, 162.46, 158.08, 136.54, 134.04, 133.01, 132.08, 131.29, 130.19, 129.61, 128.41, 115.56, 72.72, 54.85, 49.64, 24.38; **HRMS** (ESI) m/z [M+H] calcd. for $C_{16}H_{14}BrClNO_2S^+$: 397,9622, found: 397,9623; 0.3 ppm error.

Supplemental References

[1] S. Aalla, G. Gilla, R. R. Anumula, K. Charagondla, P. R. Vummenthala, P. R. Padi, *Org. Process. Res. Dev.* **2012**, *16*, 1523-1526
[2] S. Song, X. Li, J. Wei, W. Wang, Y. Zhang, L. Ai, Y. Zhu, X. Shi, X. Zhang, N. Jiao, *Nat. Catal.* **2020**, *3*, 107-115.
[3] V. Krasovskaya, A. Krasovskiy, B. H. Lipshutz, *Chem.- Asian J.* **2011**, *6*, 1974-1976.

HPLC-MS chromatogram: PMS/NaCl

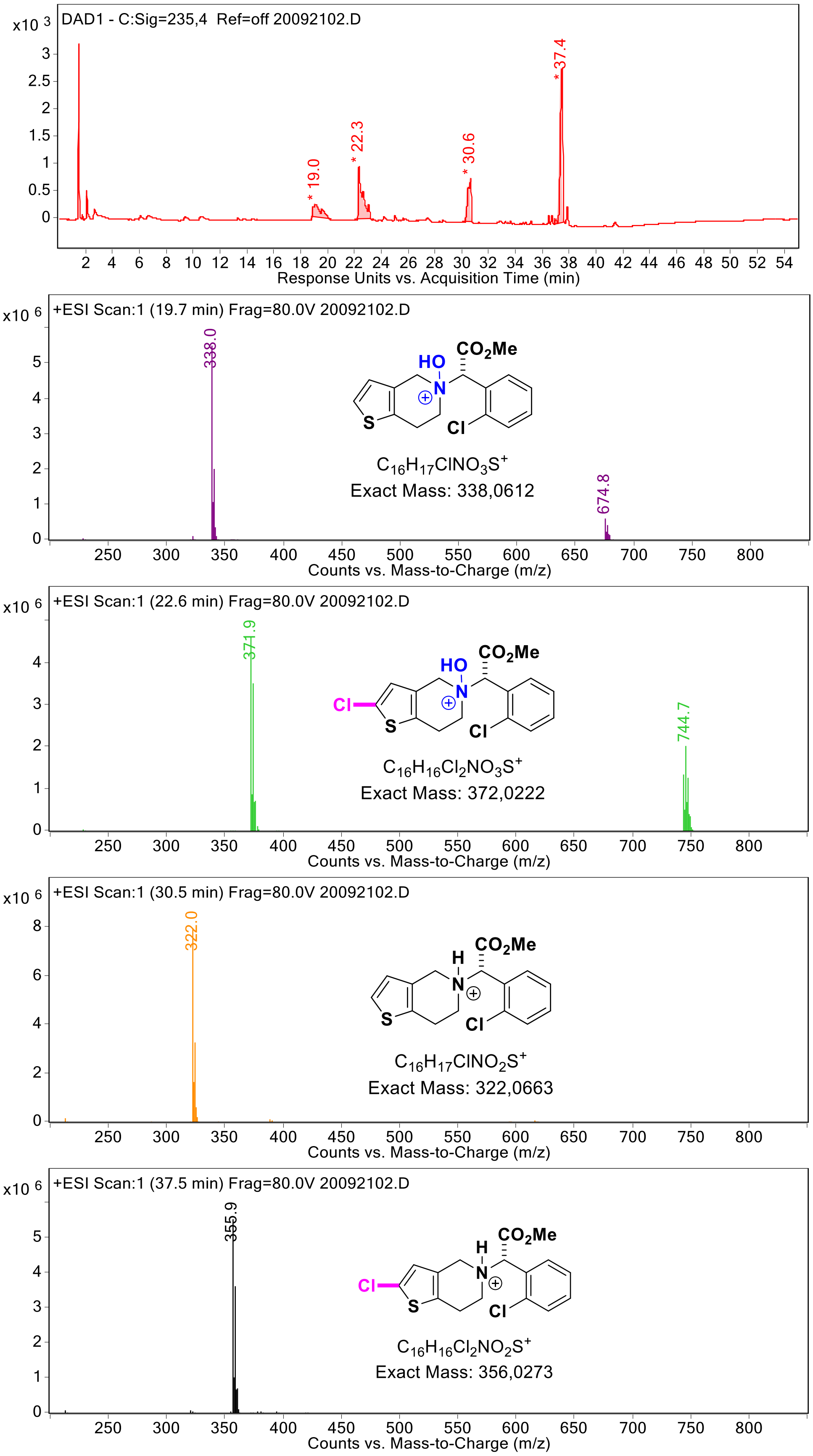

Figure S1. HPLC-MS chromatogram of mixtures (see Table 1, Entry 8).

HPLC chromatograms

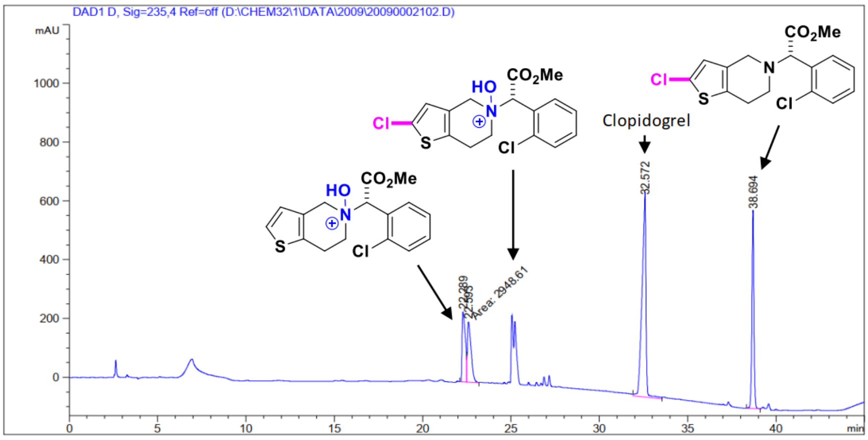

Figure S2. HPLC chromatogram (UV 235 nm) of mixtures (Table 1, Entry 8).

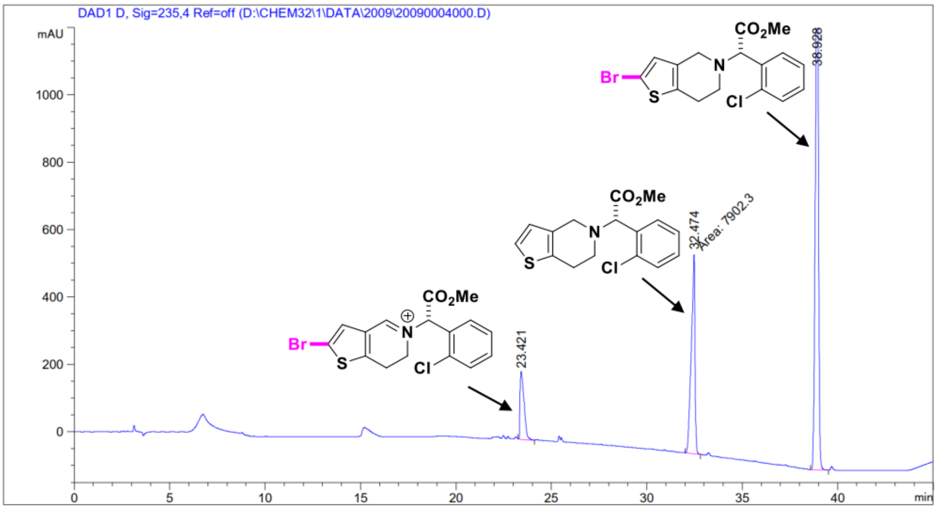

Figure S3. HPLC chromatogram (UV 235 nm) of mixtures (Table 2, Entry 4).

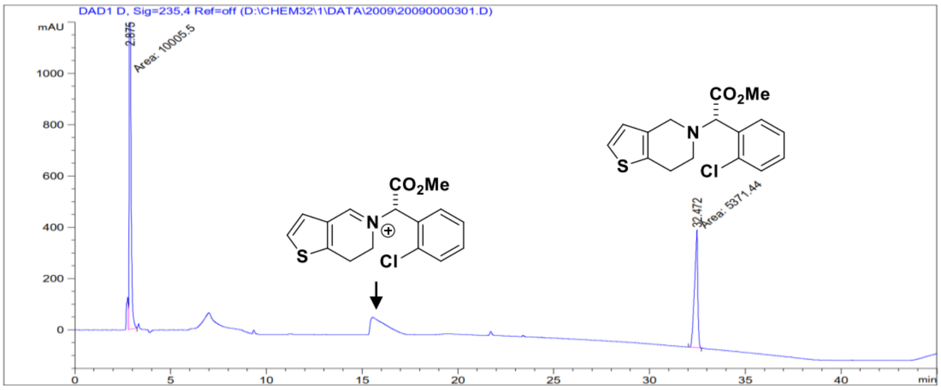

Figure S4. HPLC chromatogram (UV 235 nm) of mixtures (see Table 3, Entry 5).

HPLC conditions for separation of the products: column (Zorbax Eclipse Plus C8, 250 x 4.6 mm, 5 µm particles). Mobile phase: (A) aqueous solution of formic acid pH 2.75 and (B) ACN; Elution mode: 0-2 min: 2% B; 2-5 min: 2 to 20% B; 5-15 min: 20% B; 15-35 min: 20 to 80% B; 35-40 min: 80% B; 40-48 min: 80 to 2% B. Detection 235 nm (DAD). Flow rate: All the products are separated very well.

Structure	UV spectrum
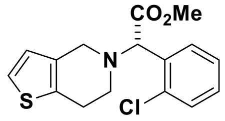	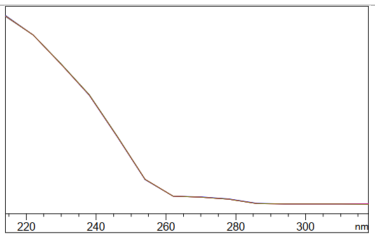
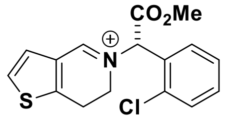	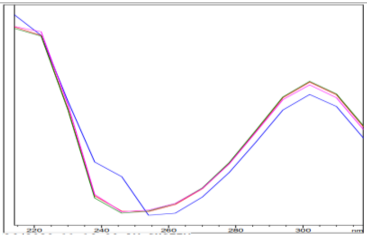
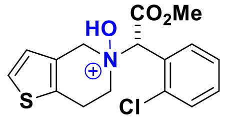	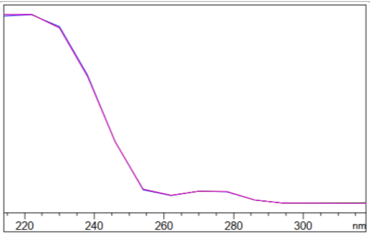
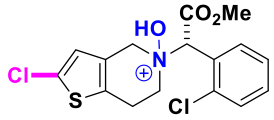	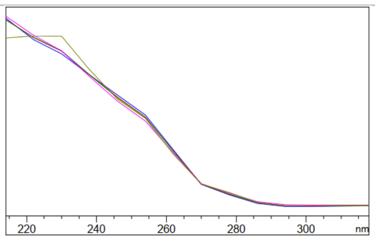
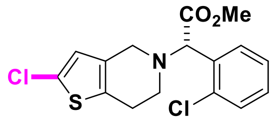	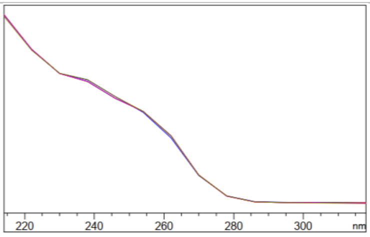
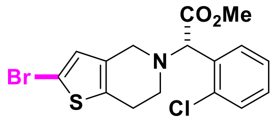	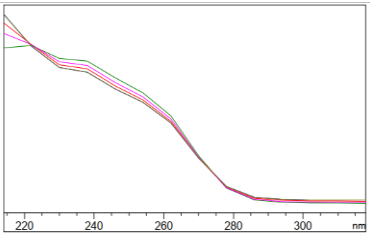
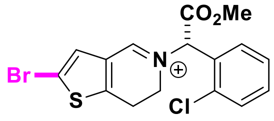	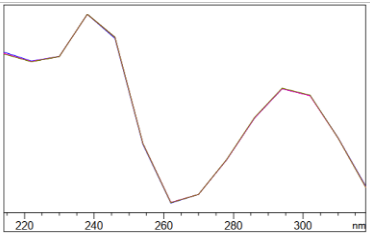

Figure S5. UV Spectrum

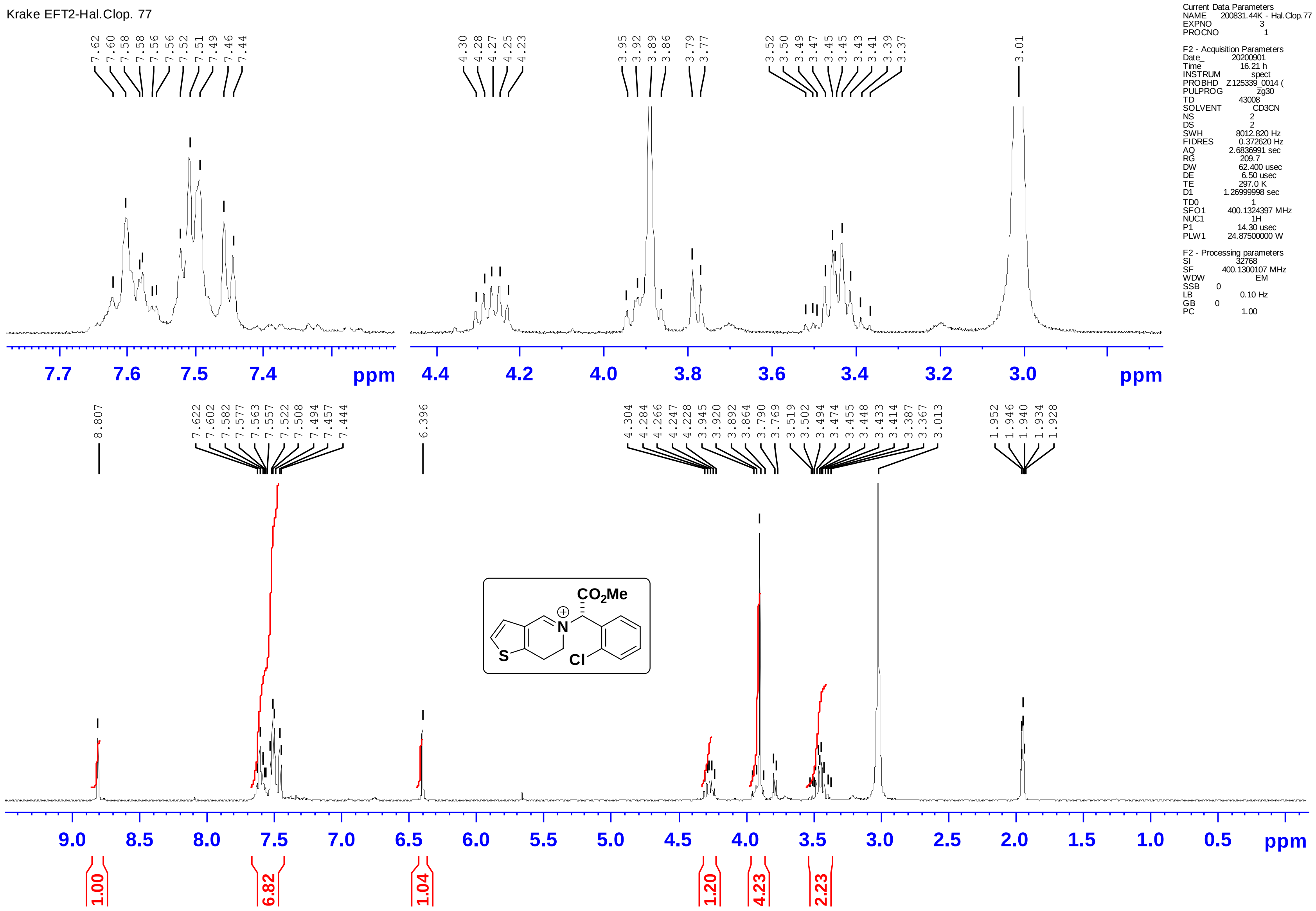

Figure S6. Copy of ^{1}H NMR spectrum of compound **DP-2**.

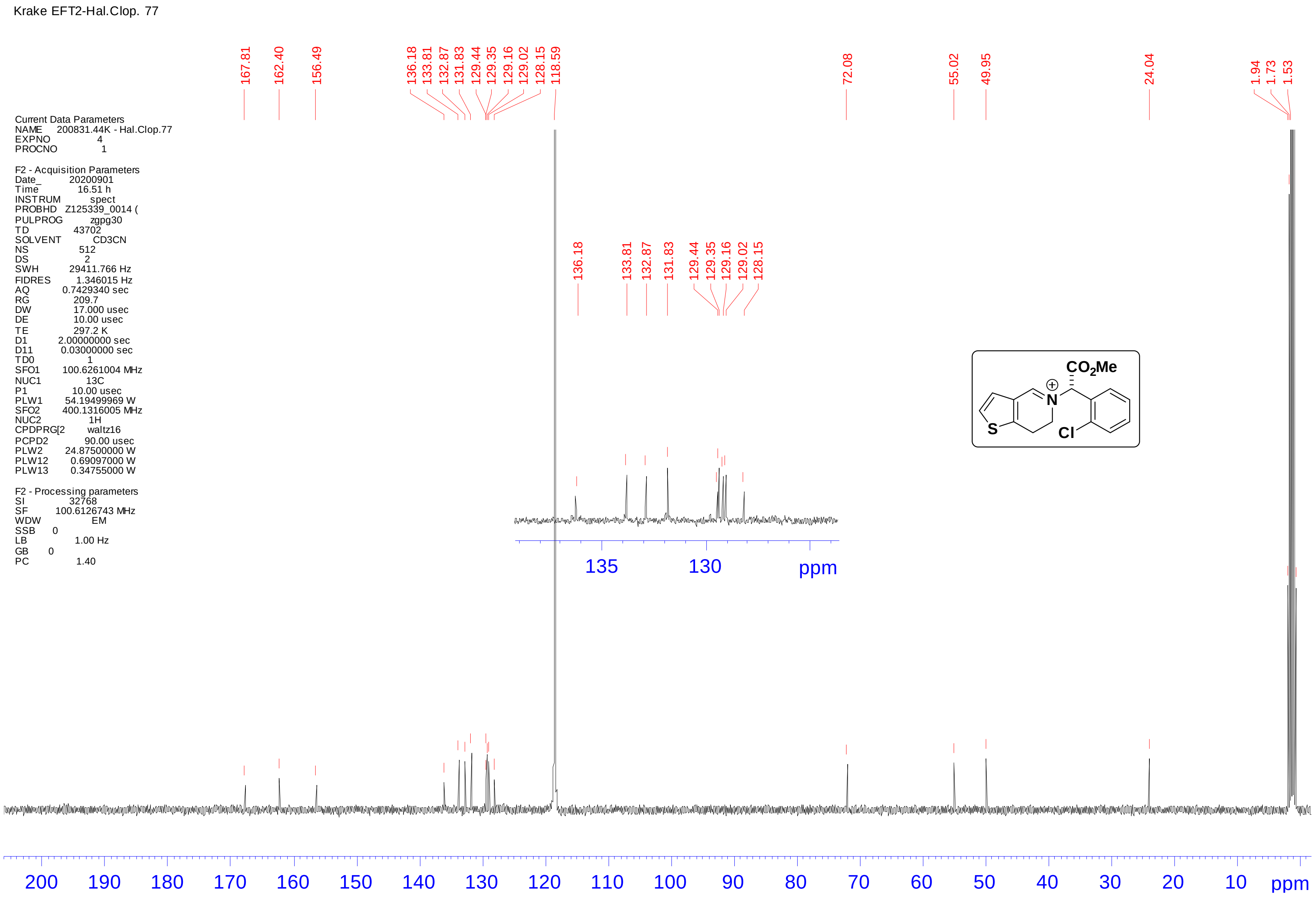

Figure S7. Copy of ^{13}C NMR spectrum of compound **DP-2**.

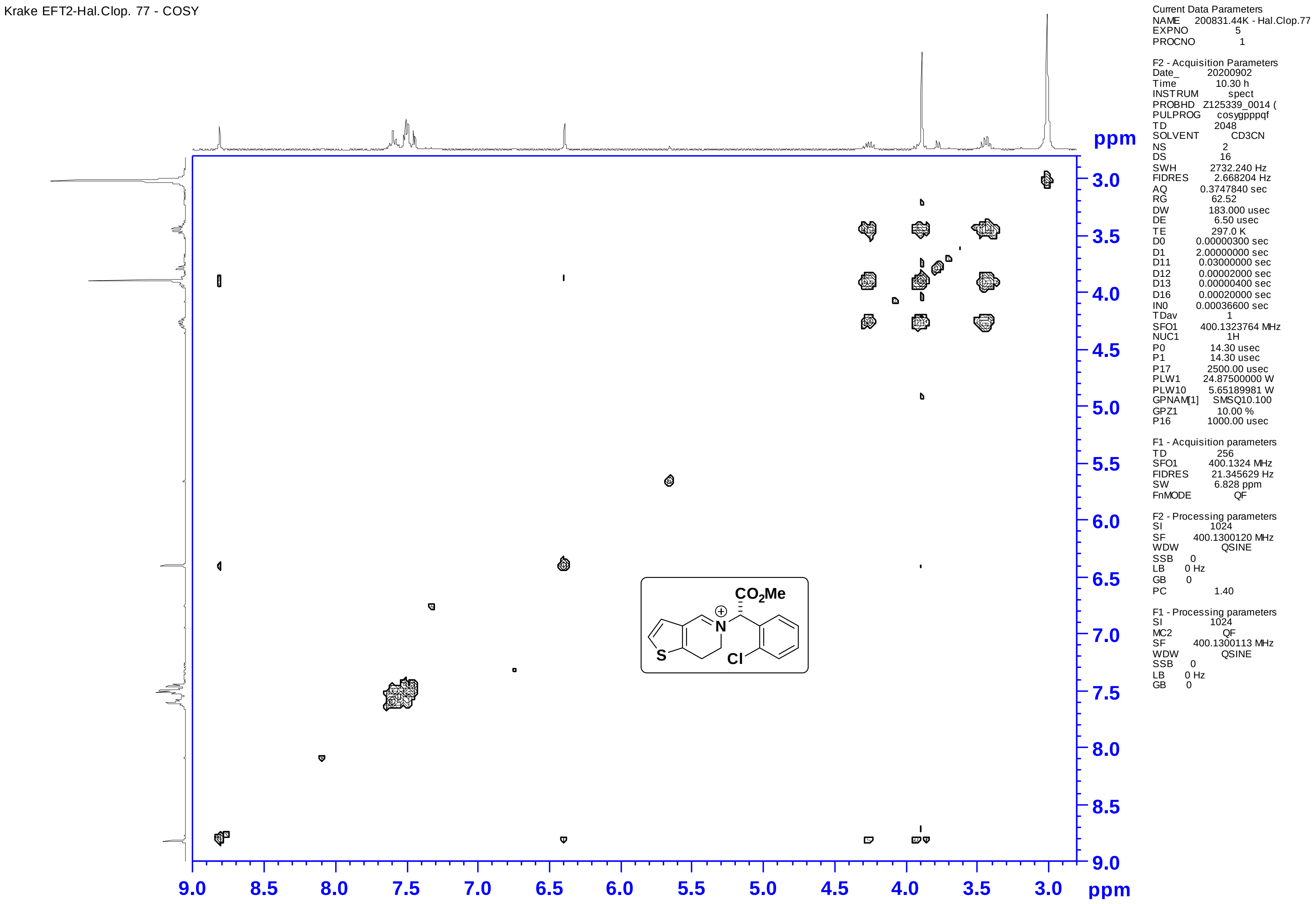

Figure S8. Copy of $^{1}H/^{1}H$ COSY spectrum of compound **DP-2**.

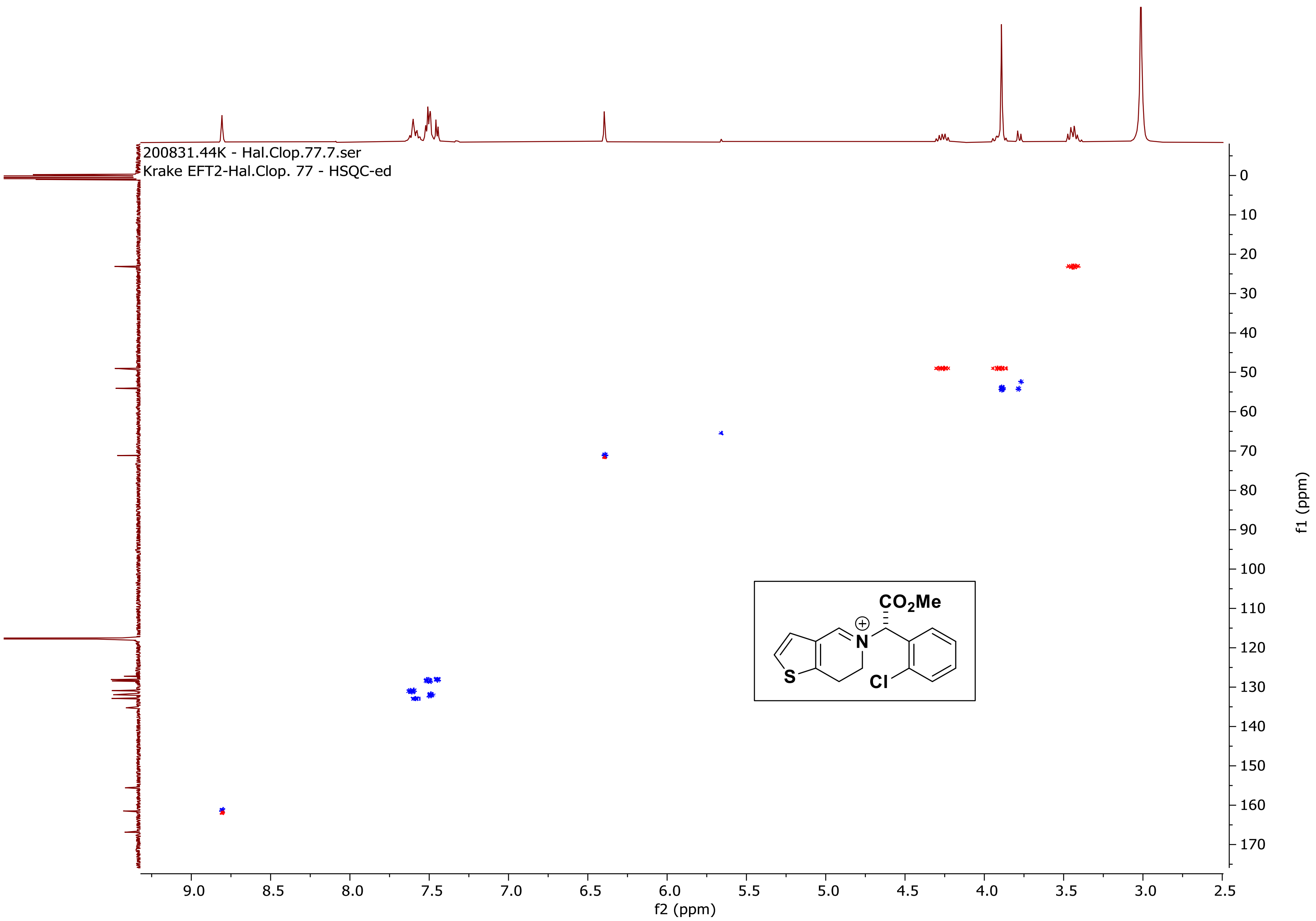

Figure S9. Copy of $^{1}H/^{13}C$ HSQC-ed. spectrum of compound **DP-2**.

Sample Report:

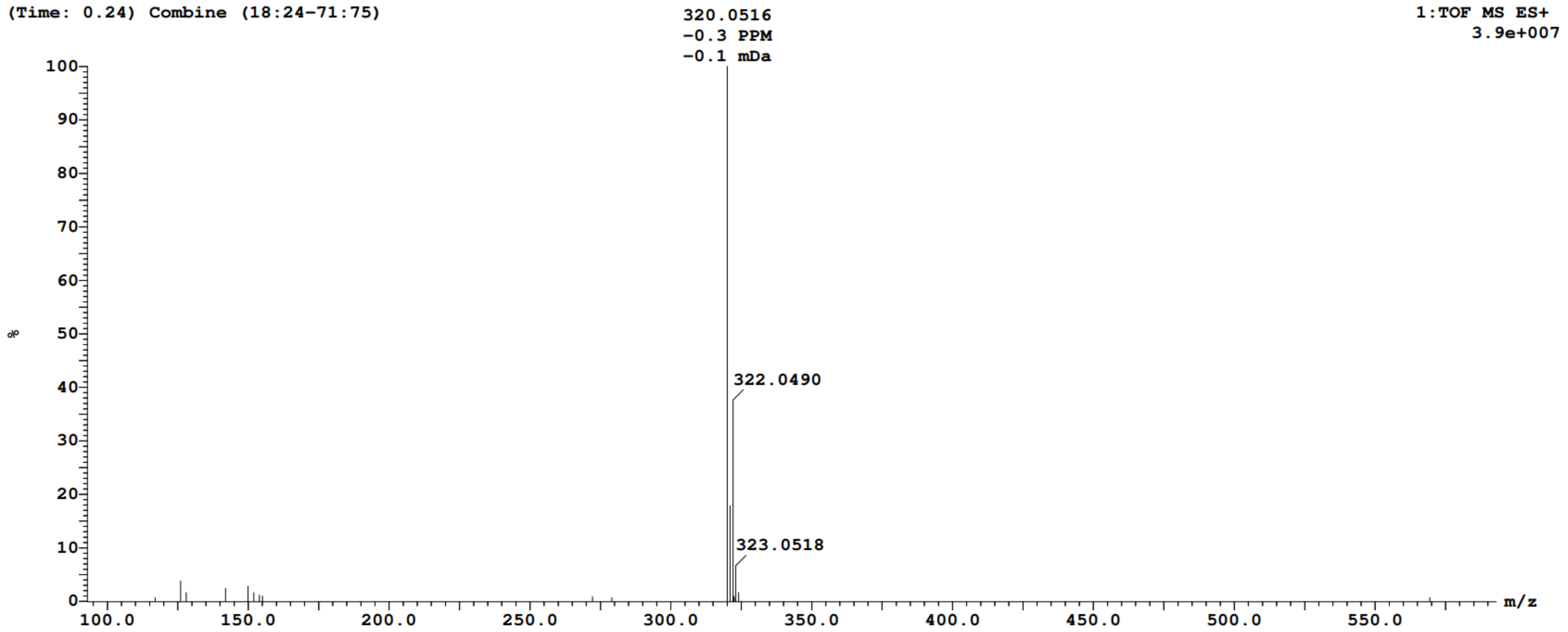

Figure S10. Copy of HRMS spectrum of compound **DP-2** ($C_{16}H_{15}ClNO_2S^+$, calc.: 320,0517, obs.: 320.0516; –0.3 ppm error).

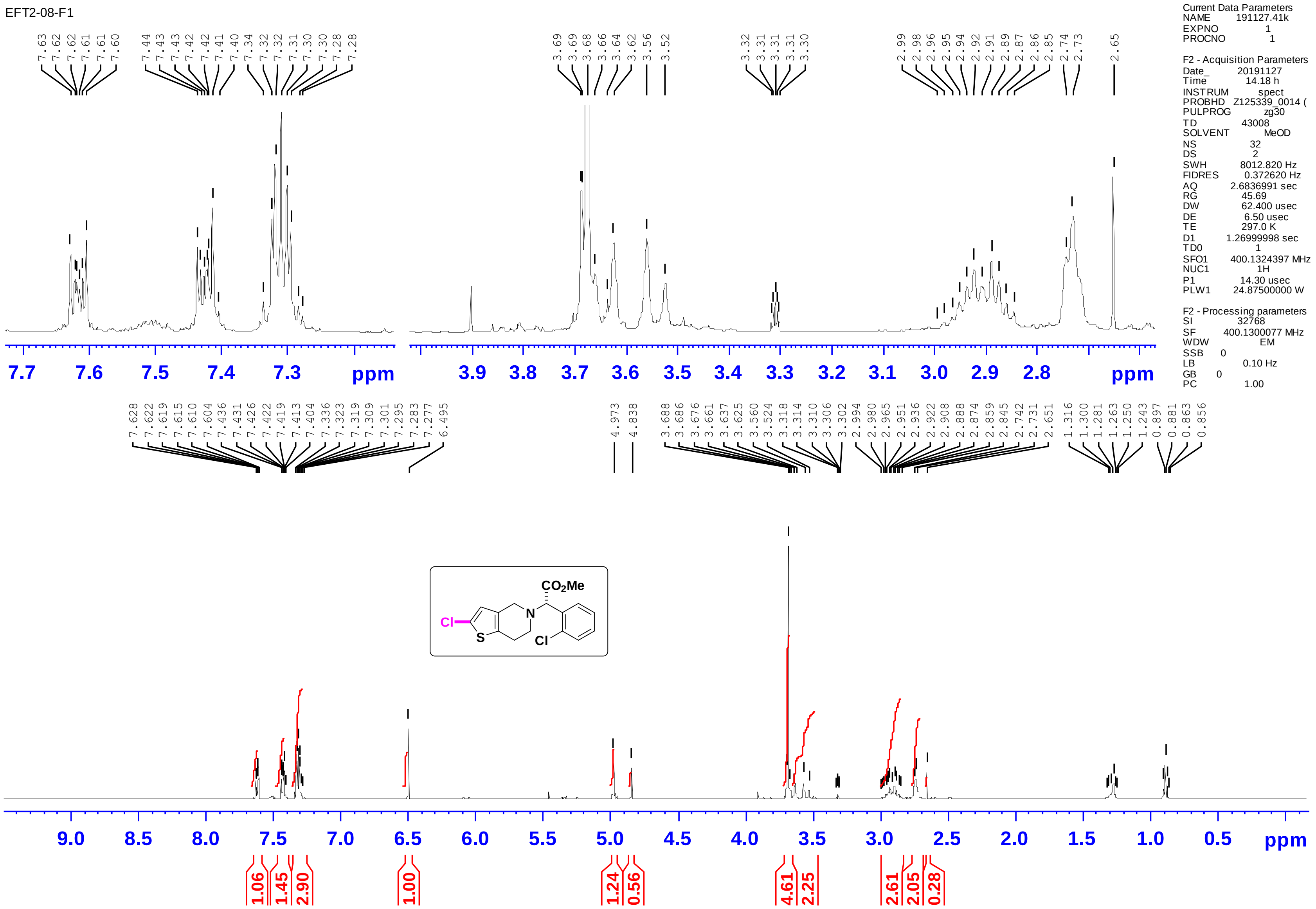

Figure S11. Copy of ^{1}H NMR spectrum of compound **DP-3a**.

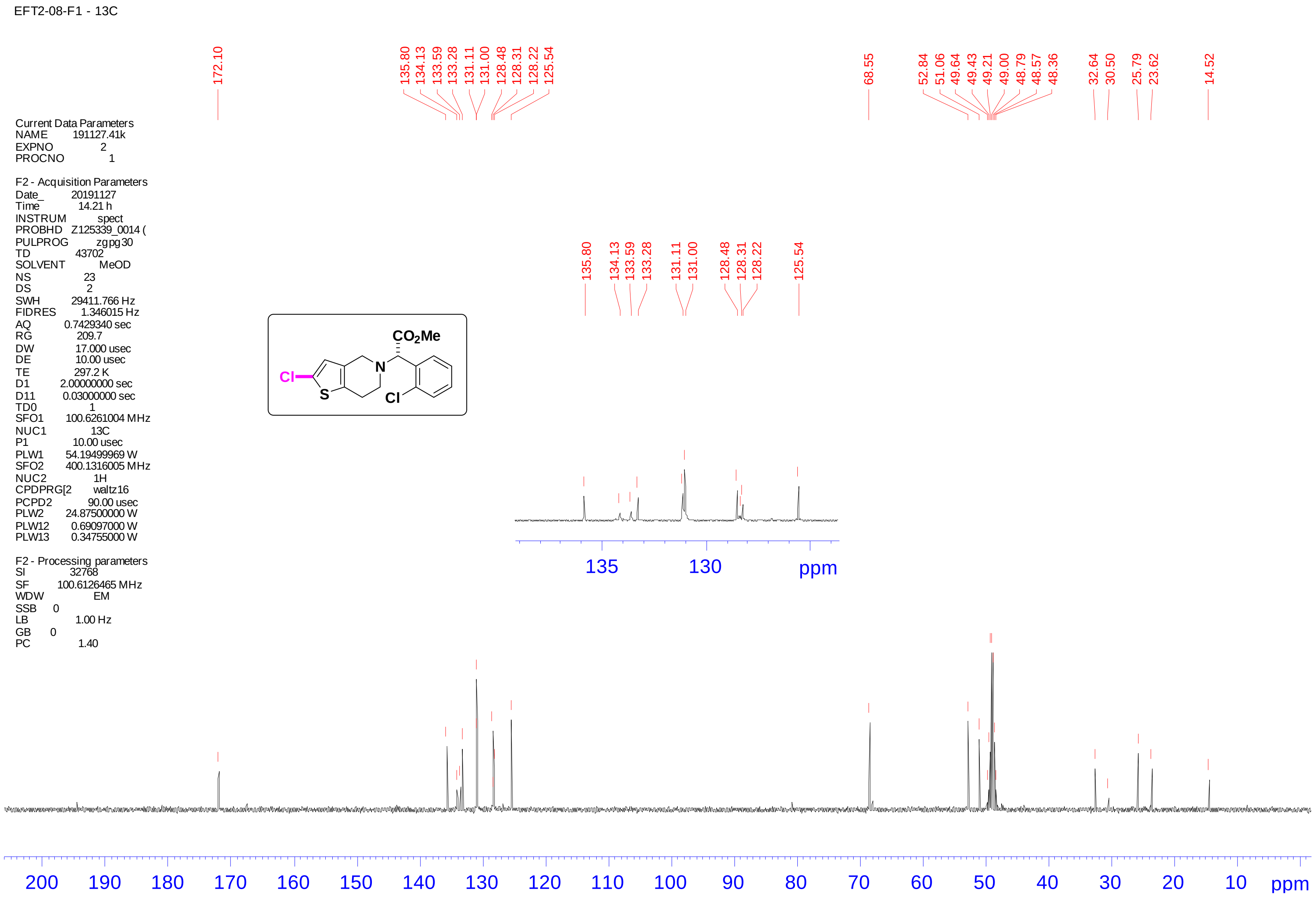

Figure S12. Copy of ^{13}C NMR spectrum of compound **DP-3a**.

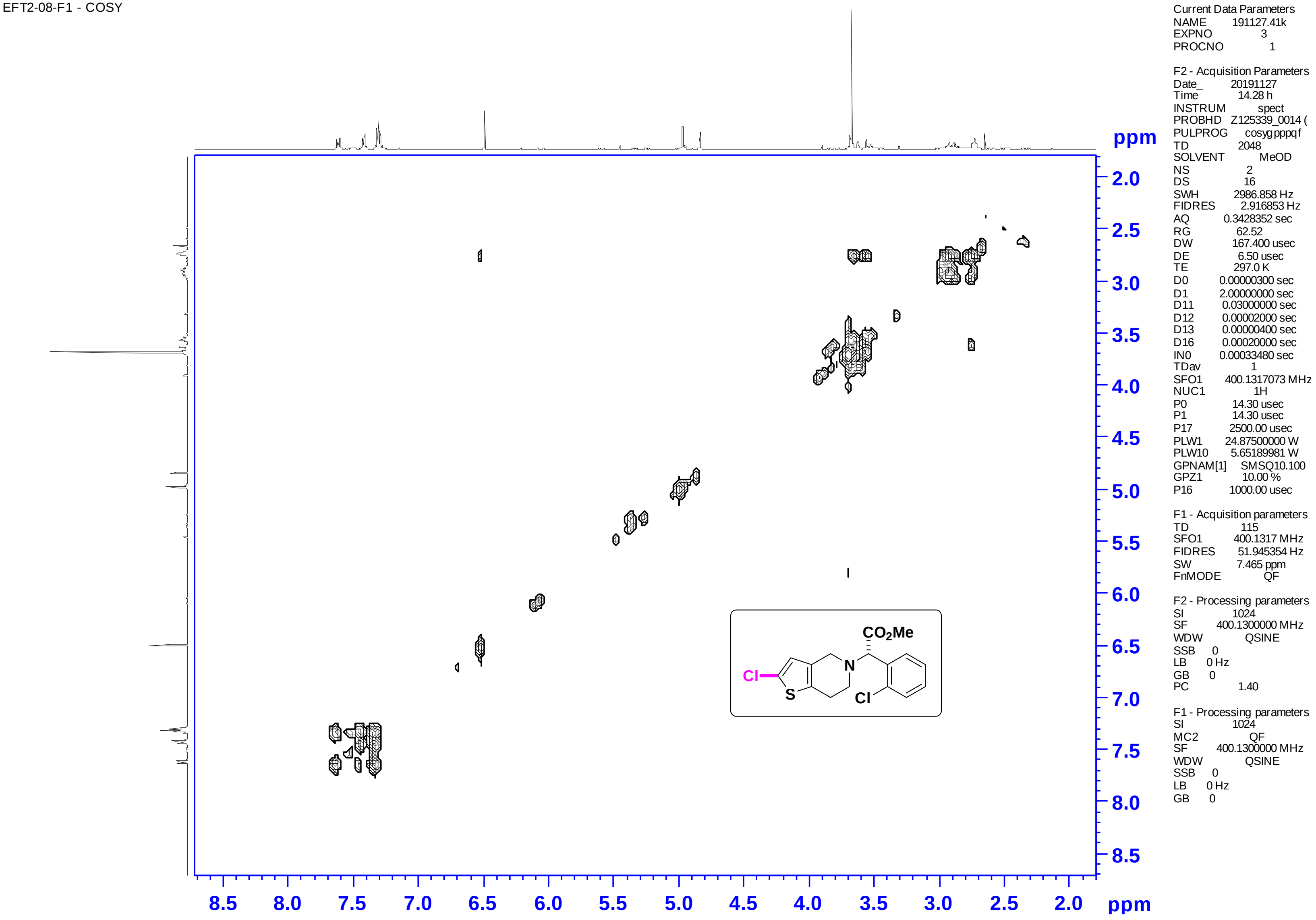

Figure S13. Copy of $^{1}H/^{1}H$ COSY spectrum of compound **DP-3a**.

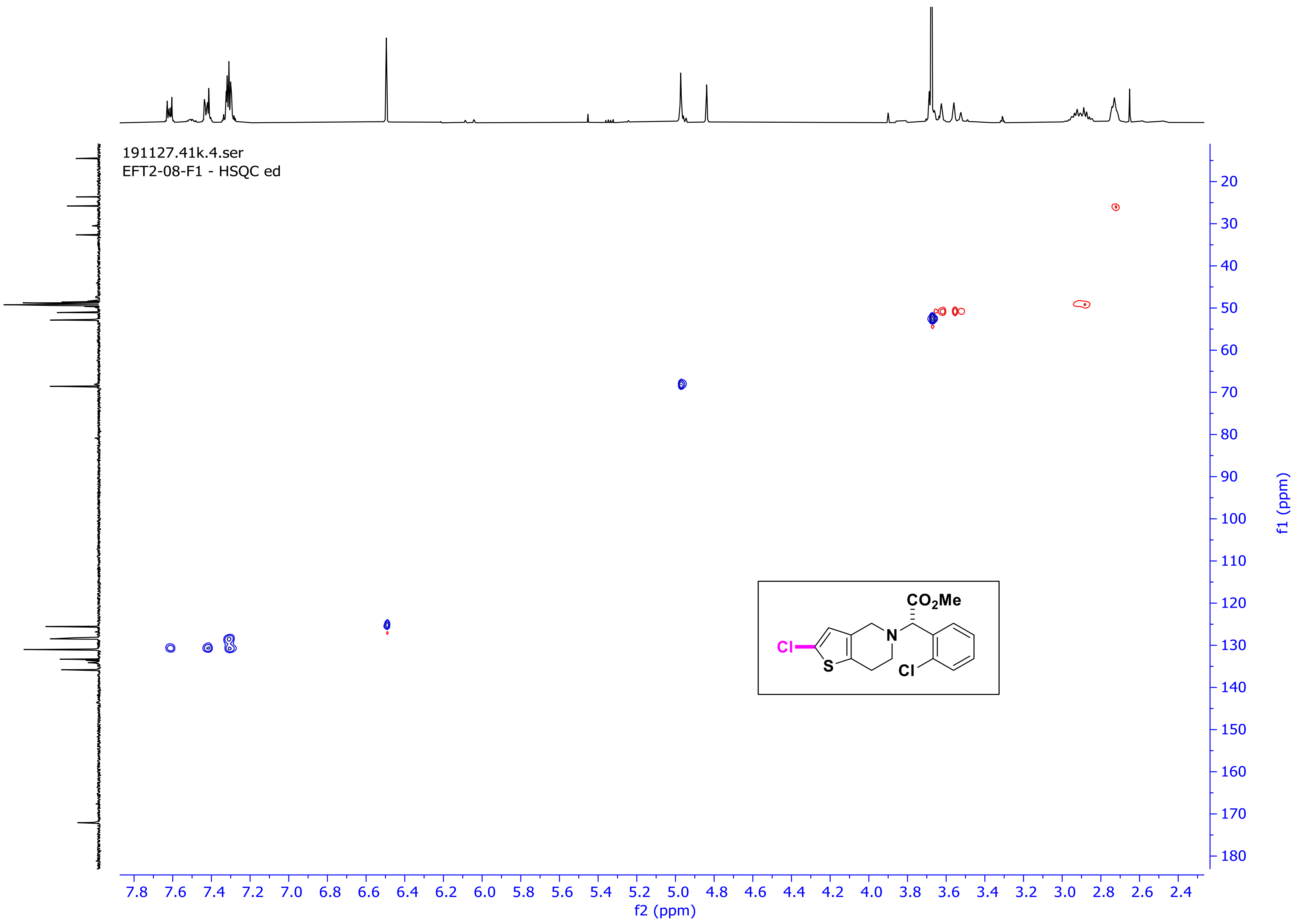

Figure S14. Copy of $^{1}H/^{13}C$ HSQC-ed spectrum of compound **DP-3a**.

EFT2-08-F1 - HMBC

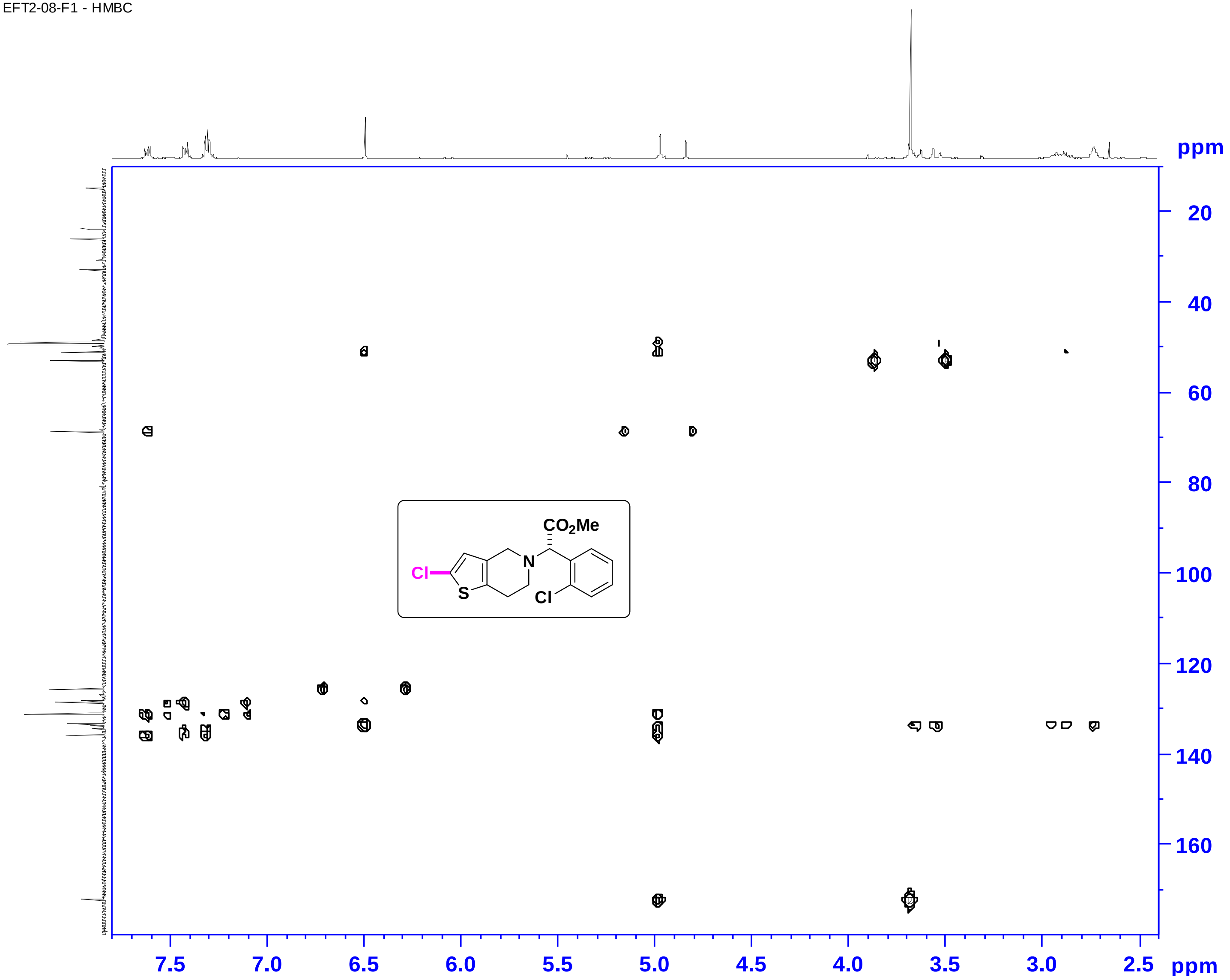

Current Data Parameters
NAME 191127.41k
EXPNO 5
PROCNO 1

F2 - Acquisition Parameters
Date_ 20191127
Time 15.28 h
INSTRUM spect
PROBHD Z125339_0014 (
PULPROG hmbcgpndqf
TD 2048
SOLVENT MeOD
NS 8
DS 16
SWH 2986.858 Hz
FIDRES 2.916853 Hz
AQ 0.3428352 sec
RG 209.7
DW 167.400 usec
DE 6.50 usec
TE 297.0 K
CNST13 8.0000000
D0 0.00000300 sec
D1 2.00000000 sec
D6 0.06250000 sec
D16 0.00020000 sec
IN0 0.00002560 sec
TDav 1
SFO1 400.1317073 MHz
NUC1 1H
P1 14.30 usec
P2 28.60 usec
PLW1 24.87500000 W
SFO2 100.6231392 MHz
NUC2 13C
P3 10.00 usec
PLW2 54.19499969 W
GPNAM[1] SMSQ10.100
GPZ1 50.00 %
GPNAM[2] SMSQ10.100
GPZ2 30.00 %
GPNAM[3] SMSQ10.100
GPZ3 40.10 %
P16 1000.00 usec

F1 - Acquisition parameters
TD 128
SFO1 100.6231 MHz
FIDRES 305.175781 Hz
SW 194.103 ppm
FnMODE QF

F2 - Processing parameters
SI 1024
SF 400.1300097 MHz
WDW SINE
SSB 0
LB 0 Hz
GB 0
PC 1.40

F1 - Processing parameters
SI 1024
MC2 QF
SF 100.6126396 MHz
WDW SINE
SSB 0
LB 0 Hz
GB 0

Figure S15. Copy of $^{1}H/^{13}C$ HMBC spectrum of compound **DP-3a**.

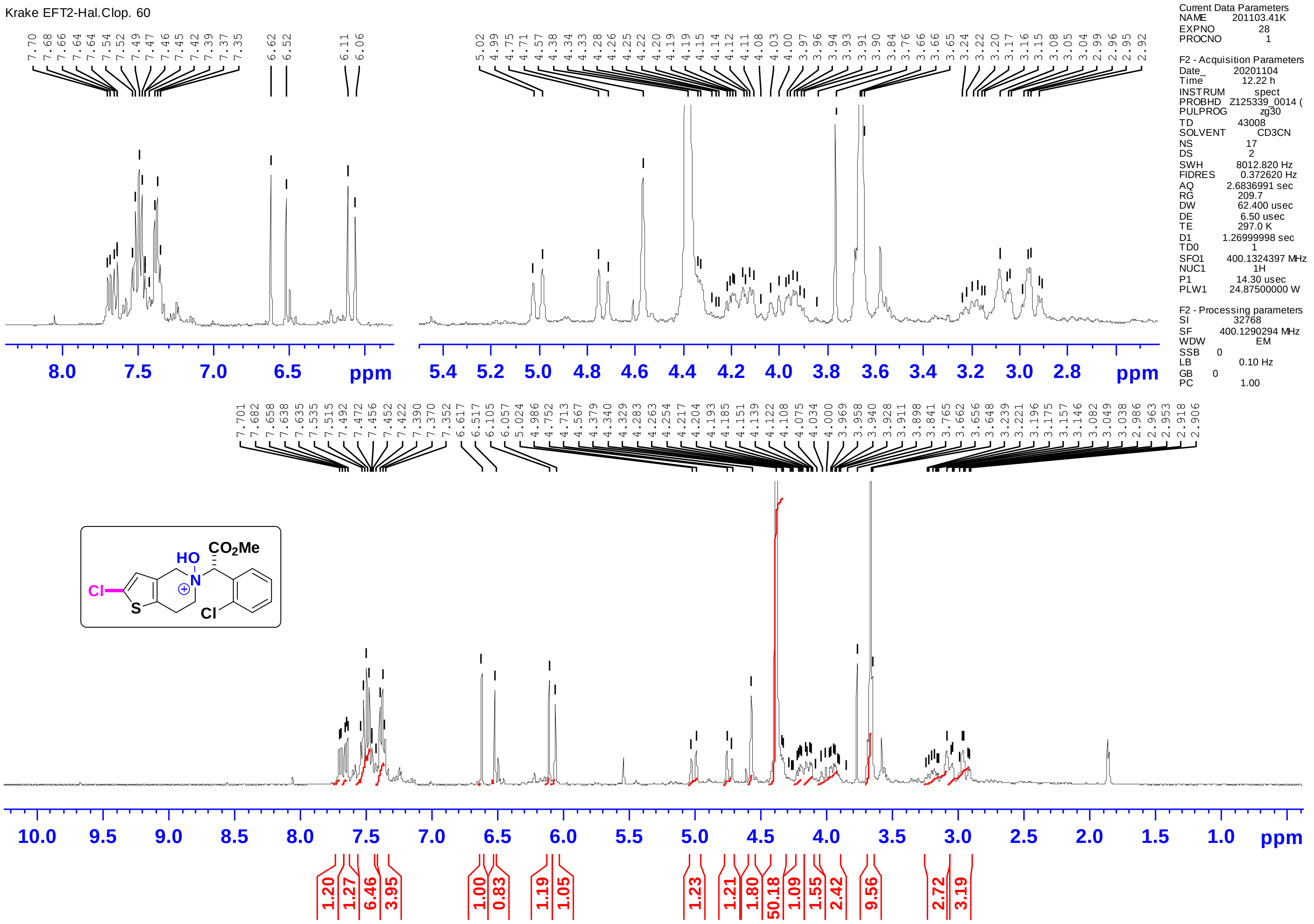

Figure S16. Copy of ^{1}H NMR spectrum of compound **DP-4a** (in a mixture D_2O/CD_3CN (2:1 v/v)).

Krake EFT2-Hal.Clop. 60

Current Data Parameters
NAME 201103.41K
EXPNO 29
PROCNO 1

F2 - Acquisition Parameters
Date_ 20201104
Time 15.55 h
INSTRUM spect
PROBHD Z125339_0014 (
PULPROG zgpg30
TD 43702
SOLVENT CD3CN
NS 4544
DS 2
SWH 29411.766 Hz
FIDRES 1.346015 Hz
AQ 0.7429340 sec
RG 209.7
DW 17.000 usec
DE 10.00 usec
TE 297.0 K
D1 2.00000000 sec
D11 0.03000000 sec
TD0 1
SFO1 100.6261004 MHz
NUC1 13C
P1 10.00 usec
PLW1 54.19499969 W
SFO2 400.1316005 MHz
NUC2 1H
CPDPRG[2 waltz16
PCPD2 90.00 usec
PLW2 24.87500000 W
PLW12 0.69097000 W
PLW13 0.34755000 W

F2 - Processing parameters
SI 32768
SF 100.6124327 MHz
WDW EM
SSB 0
LB 1.00 Hz
GB 0
PC 1.40

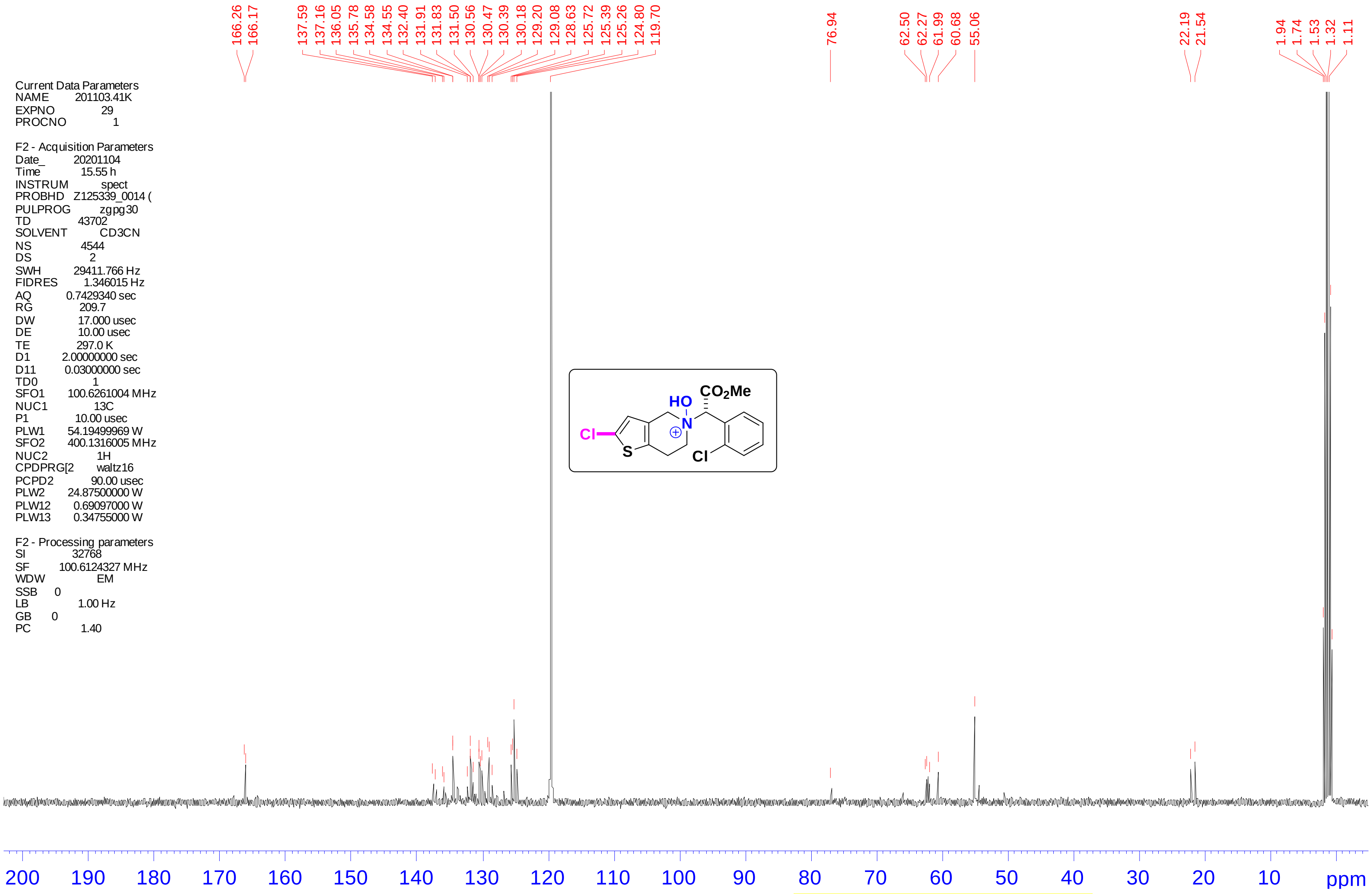

Figure S17. Copy of ^{13}C NMR spectrum of compound **DP-4a** (in a mixture D_2O/CD_3CN (2:1 v/v)).

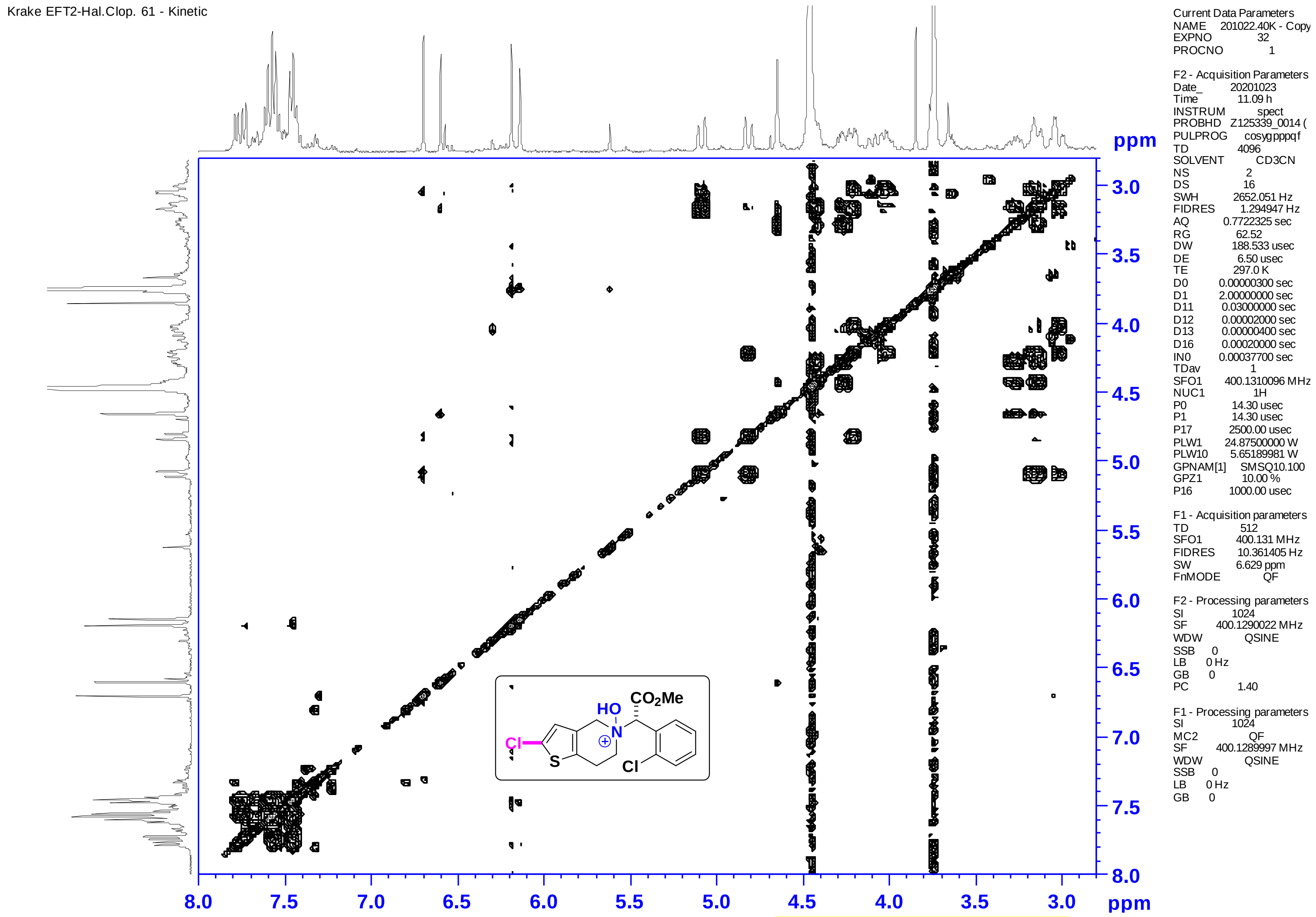

Figure S18. Copy of $^{1}H/^{1}H$ COSY spectrum of compound **DP-4a** (in a mixture D_2O/CD_3CN (2:1 v/v)).

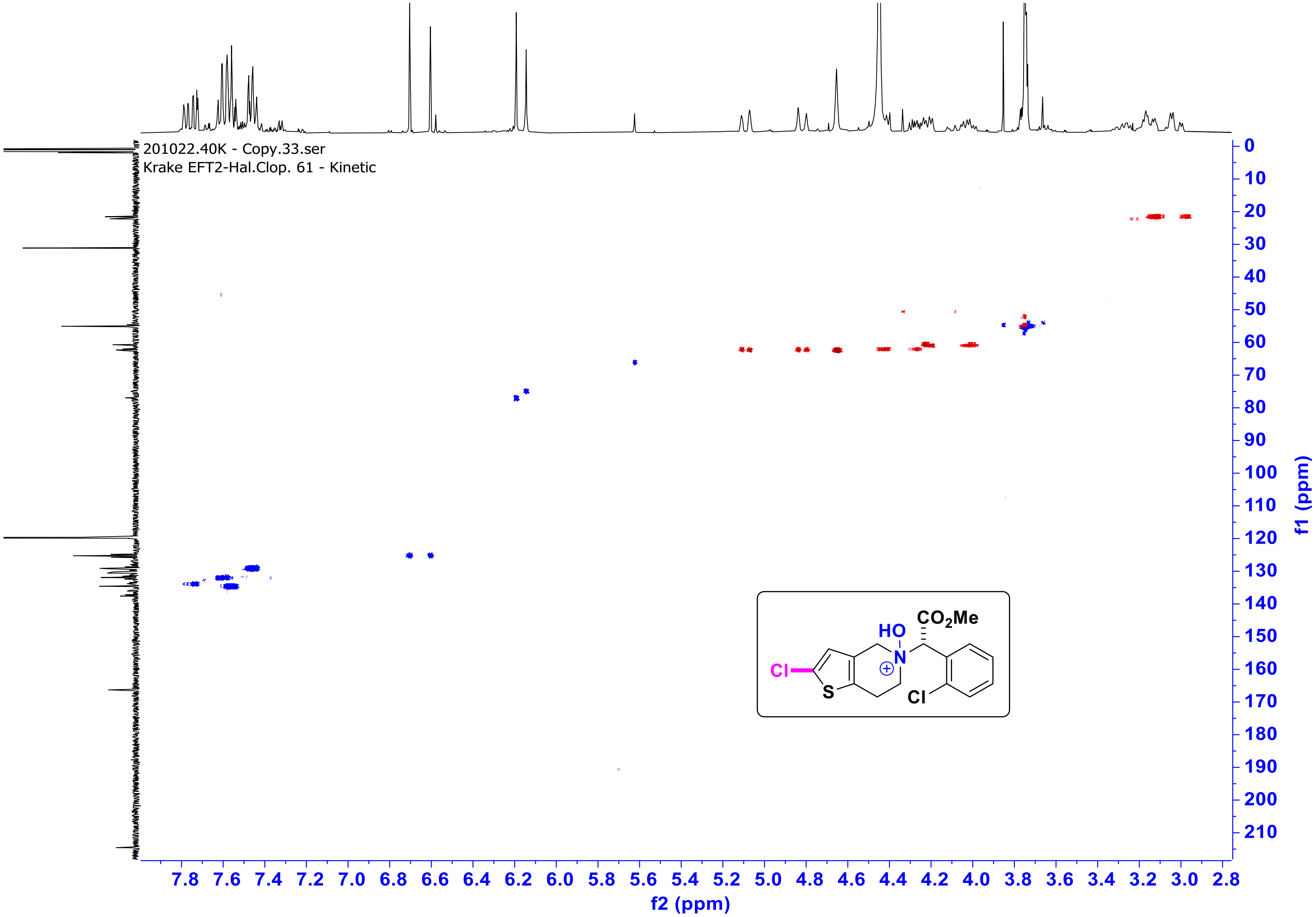

Figure S19. Copy of $^1H/^{13}C$ HSQC-ed. spectrum of compound **DP-4a** (in a mixture D_2O/CD_3CN (2:1 v/v)).

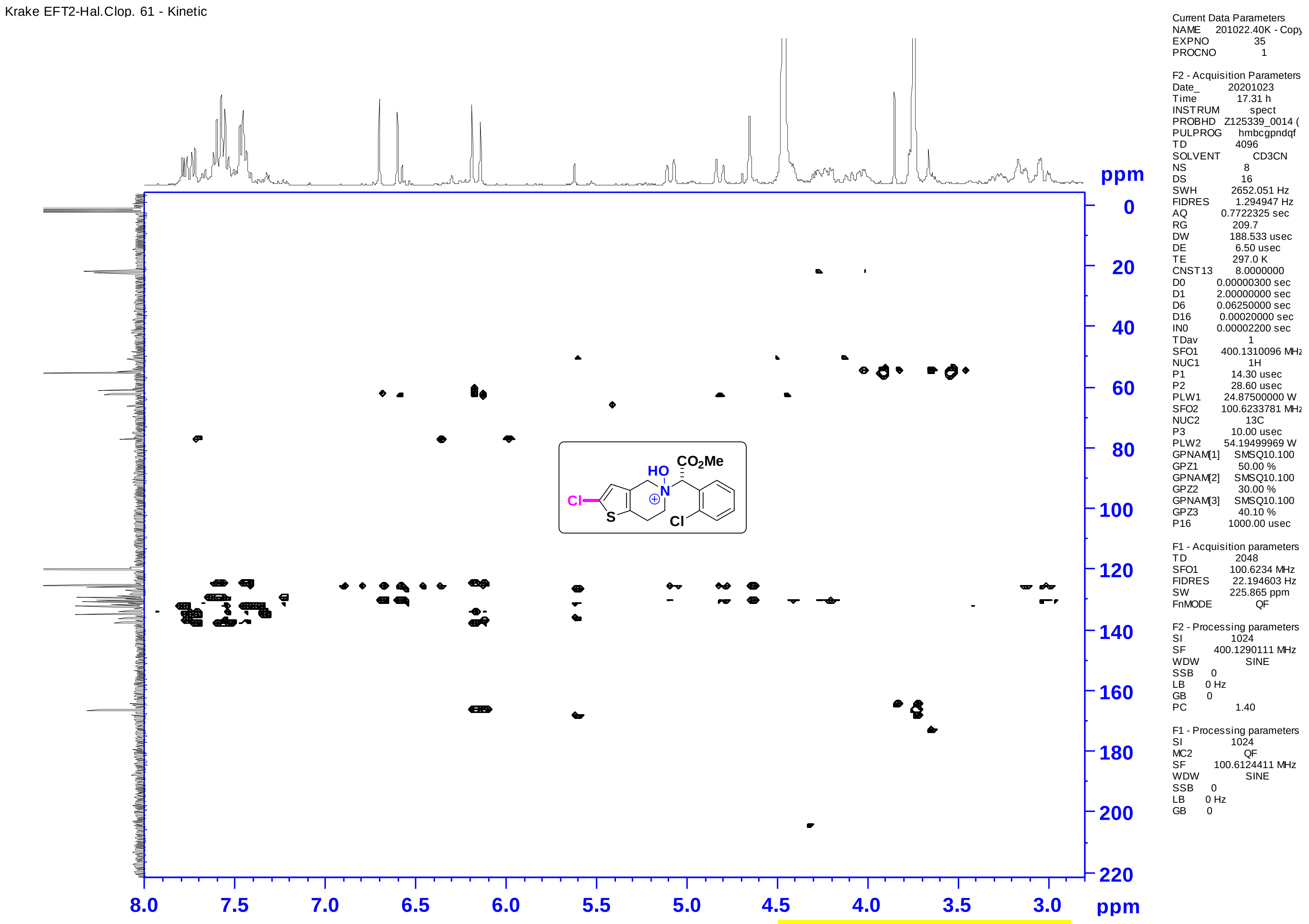

Figure S20. Copy of ^{1}H/^{13}C HMBC spectrum of compound **DP-4a** (in a mixture D_2O/CD_3CN (2:1 v/v)).

Sample Report:

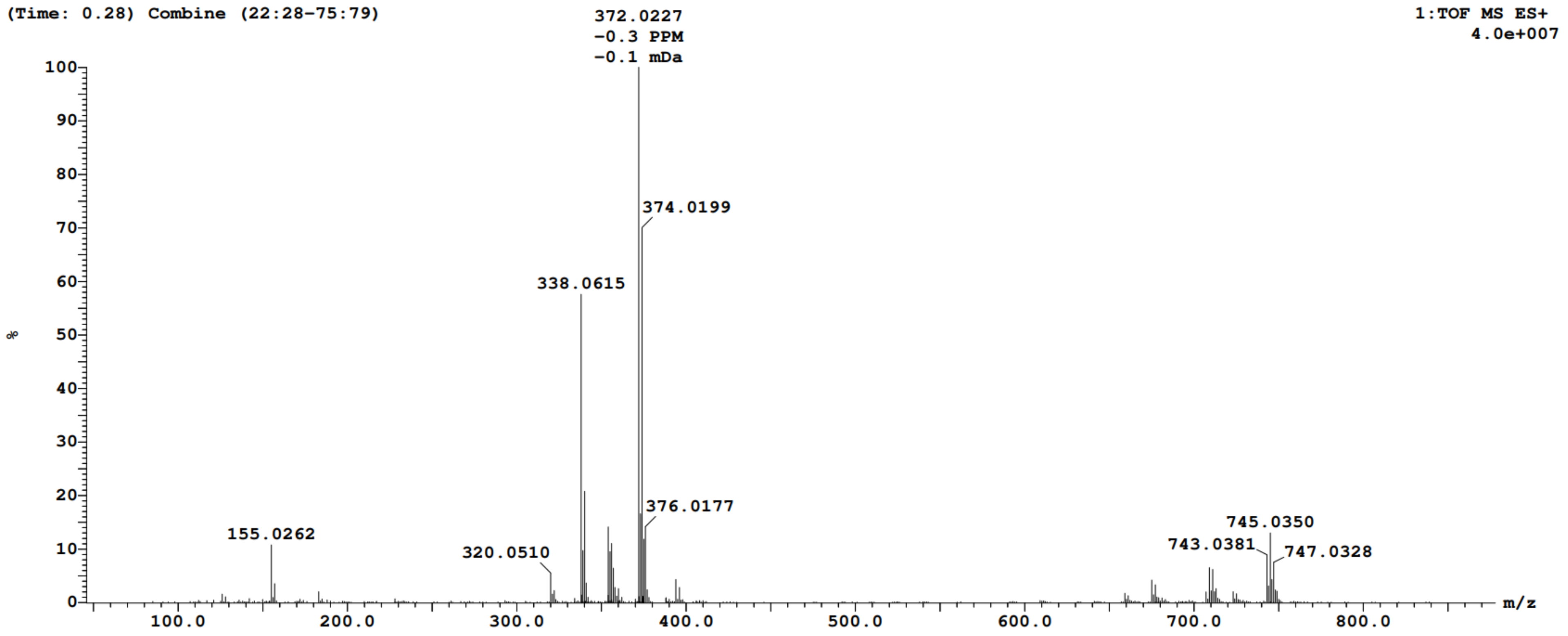

Figure S21. Copy of HRMS spectrum of compound **DP-4a** ($C_{16}H_{15}O_3NSCl_2$, calc. $(M+1)^+$: 372.0228 found: 372.0227; –0.3 ppm error).

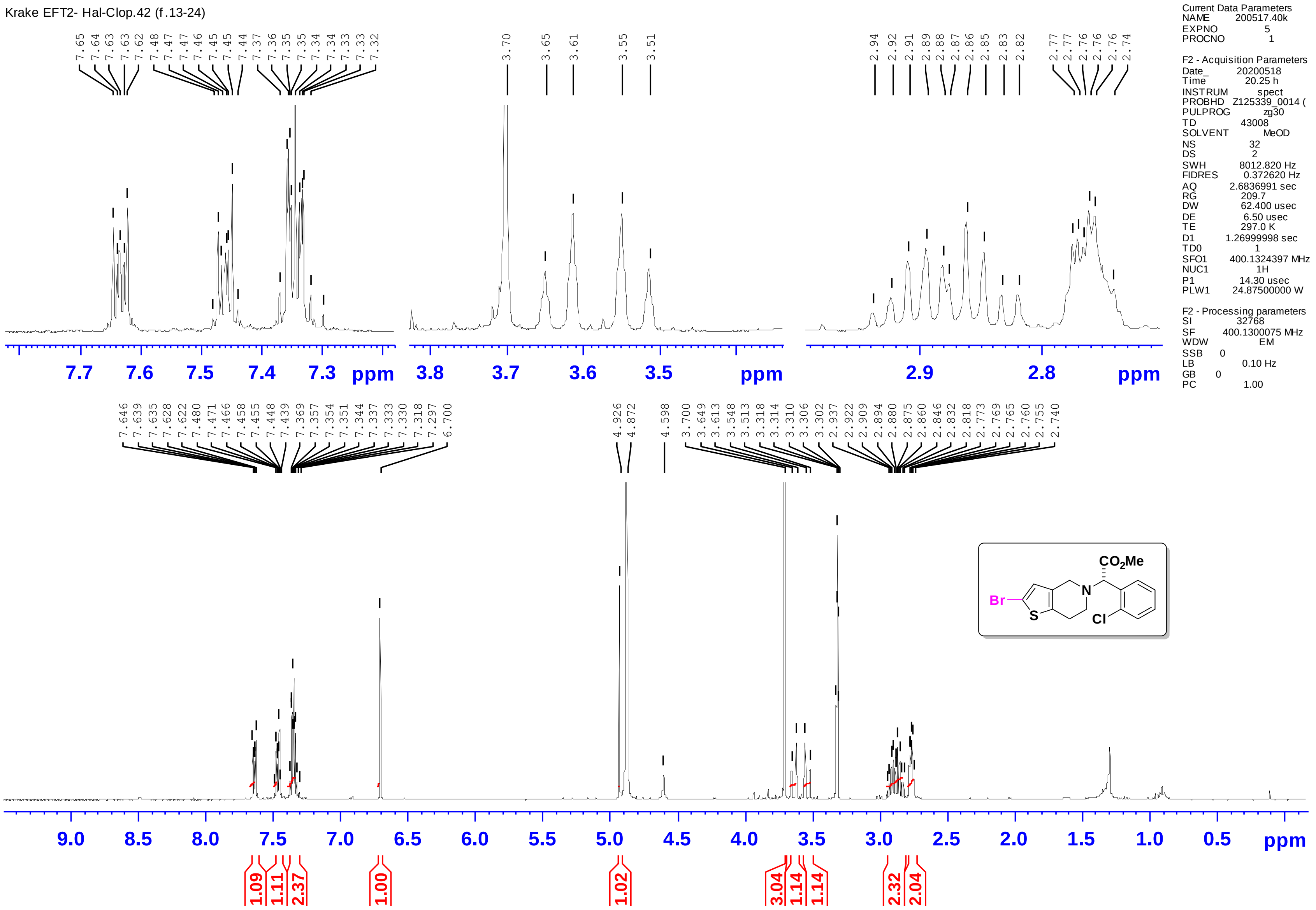

Figure S22. Copy of ^{1}H NMR spectrum of compound **DP-3b**.

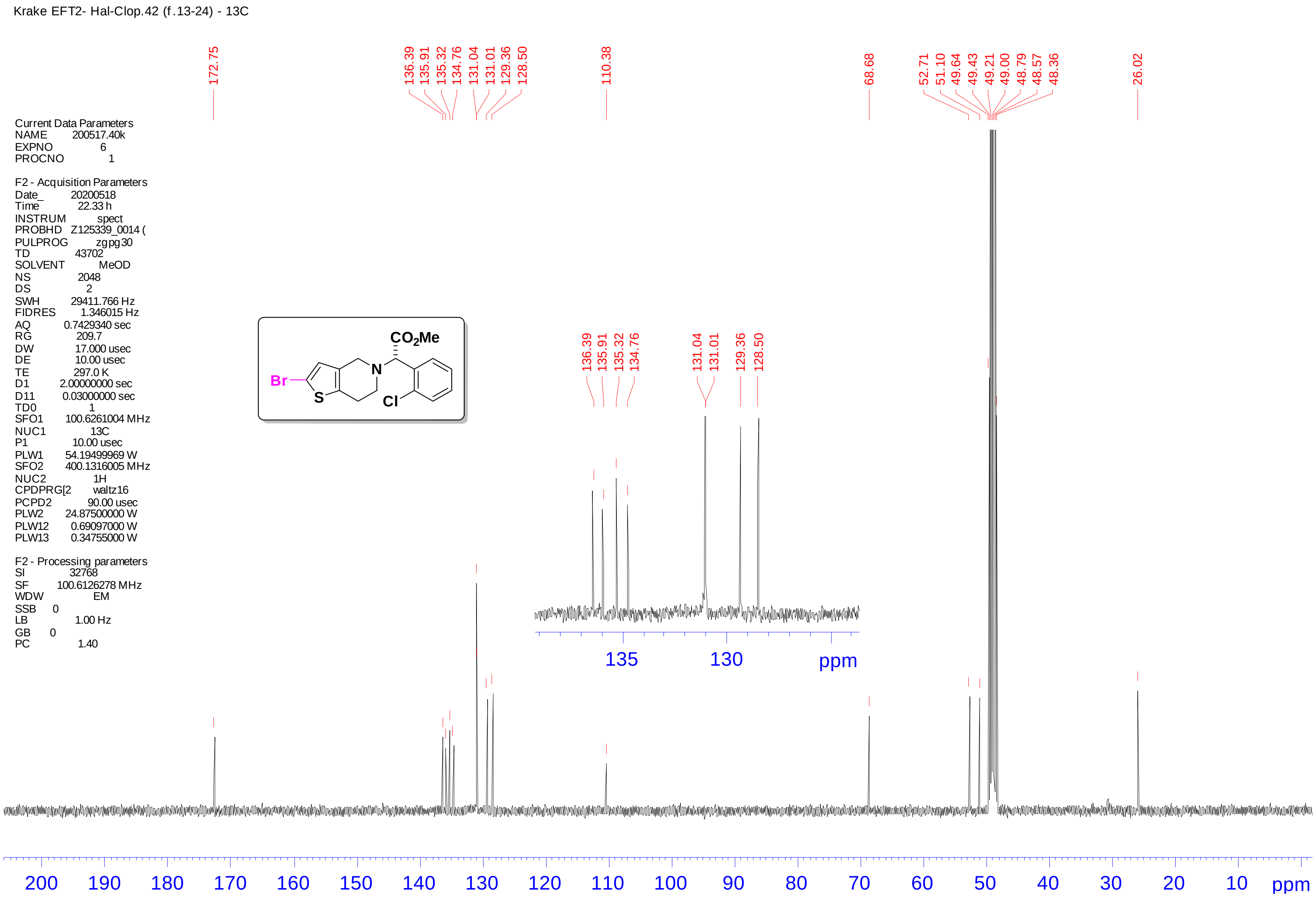

Figure S23. Copy of ^{13}C NMR spectrum of compound **DP-3b**.

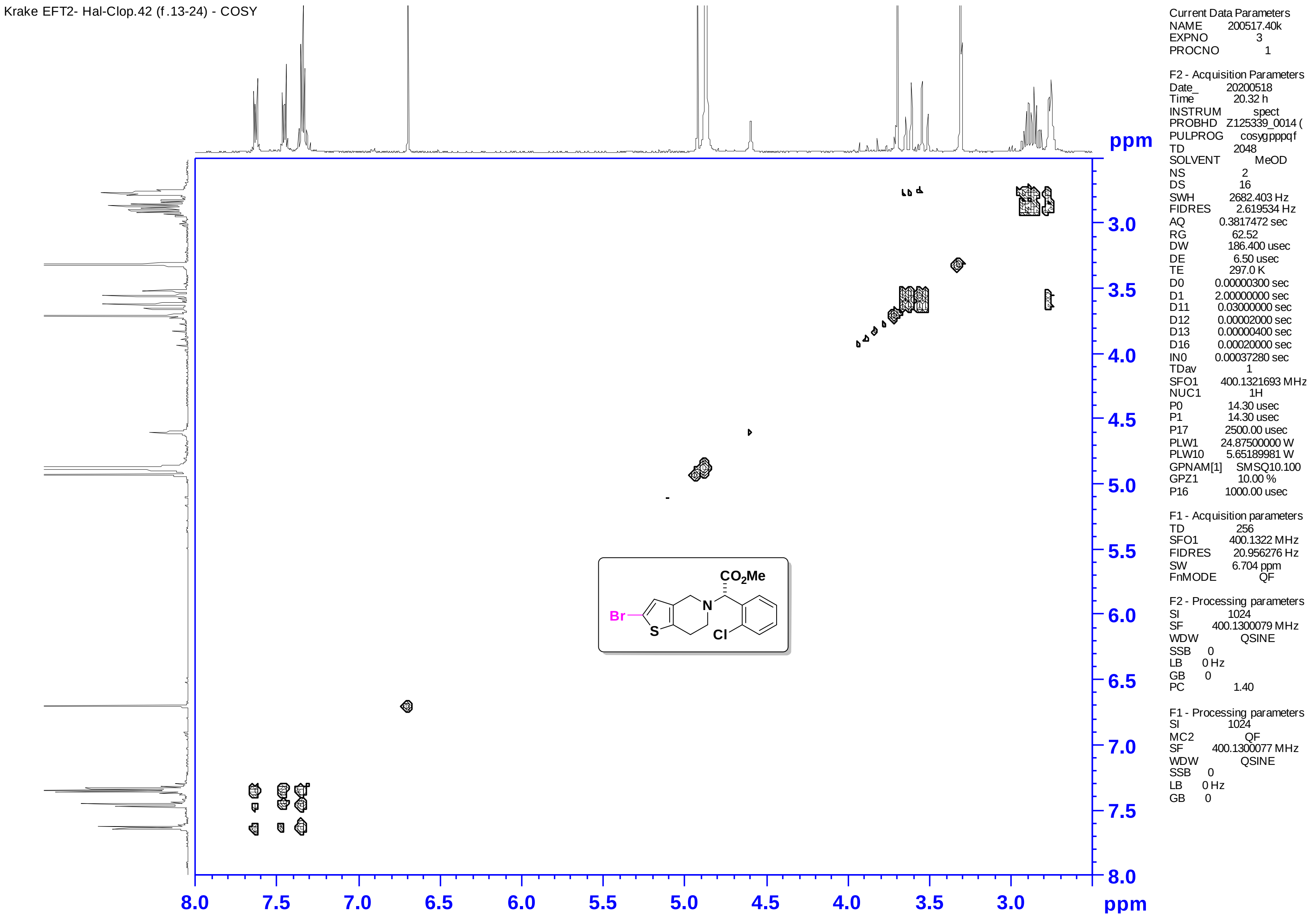

Figure S24. Copy of $^{1}H/^{1}H$ COSY spectrum of compound **DP-3b**.

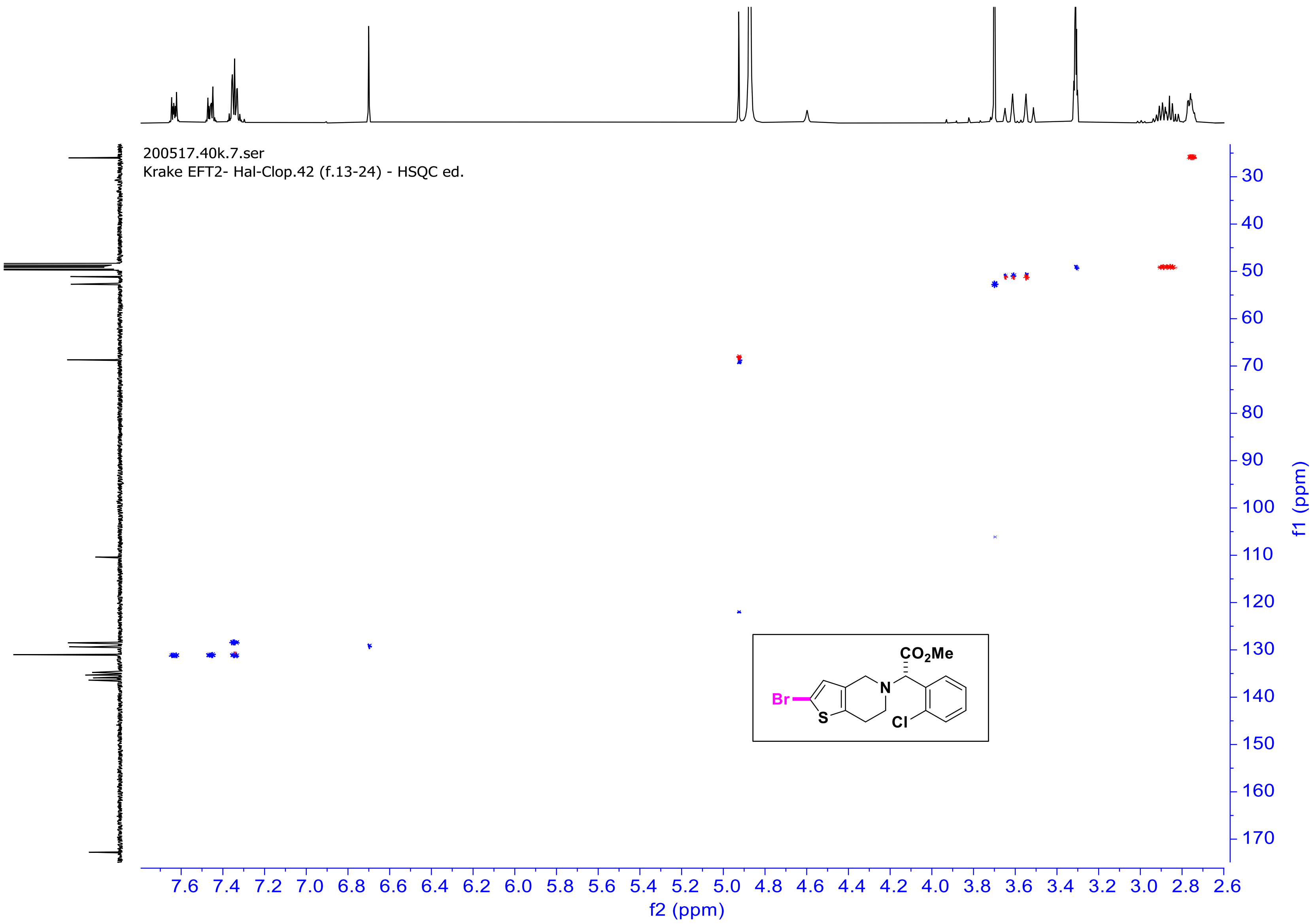

Figure S25. Copy of $^1H/^{13}C$ HSQC-ed. spectrum of compound **DP-3b**.

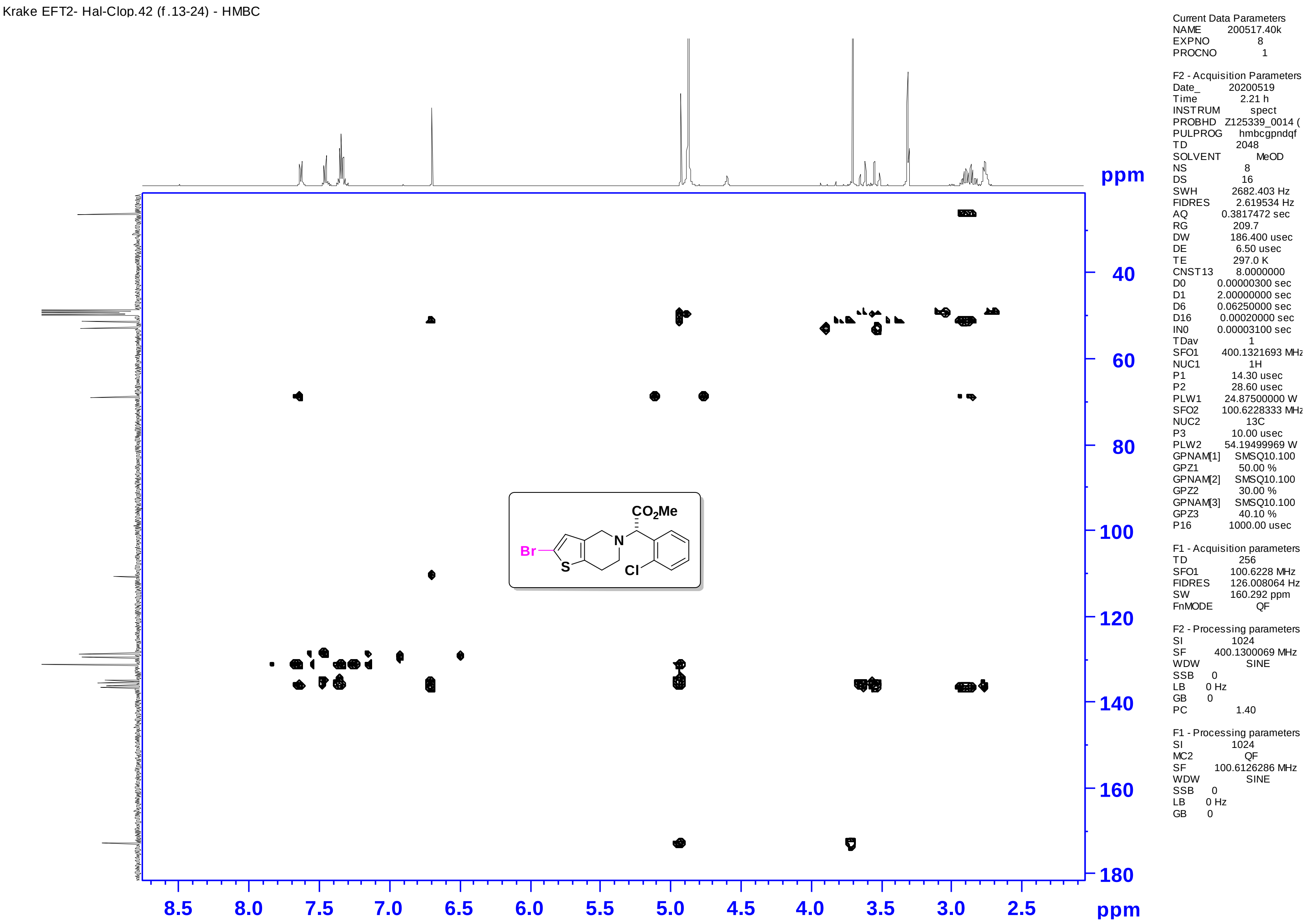

Figure S26. Copy of $^{1}H/^{13}C$ HMBC spectrum of compound **DP-3b**.

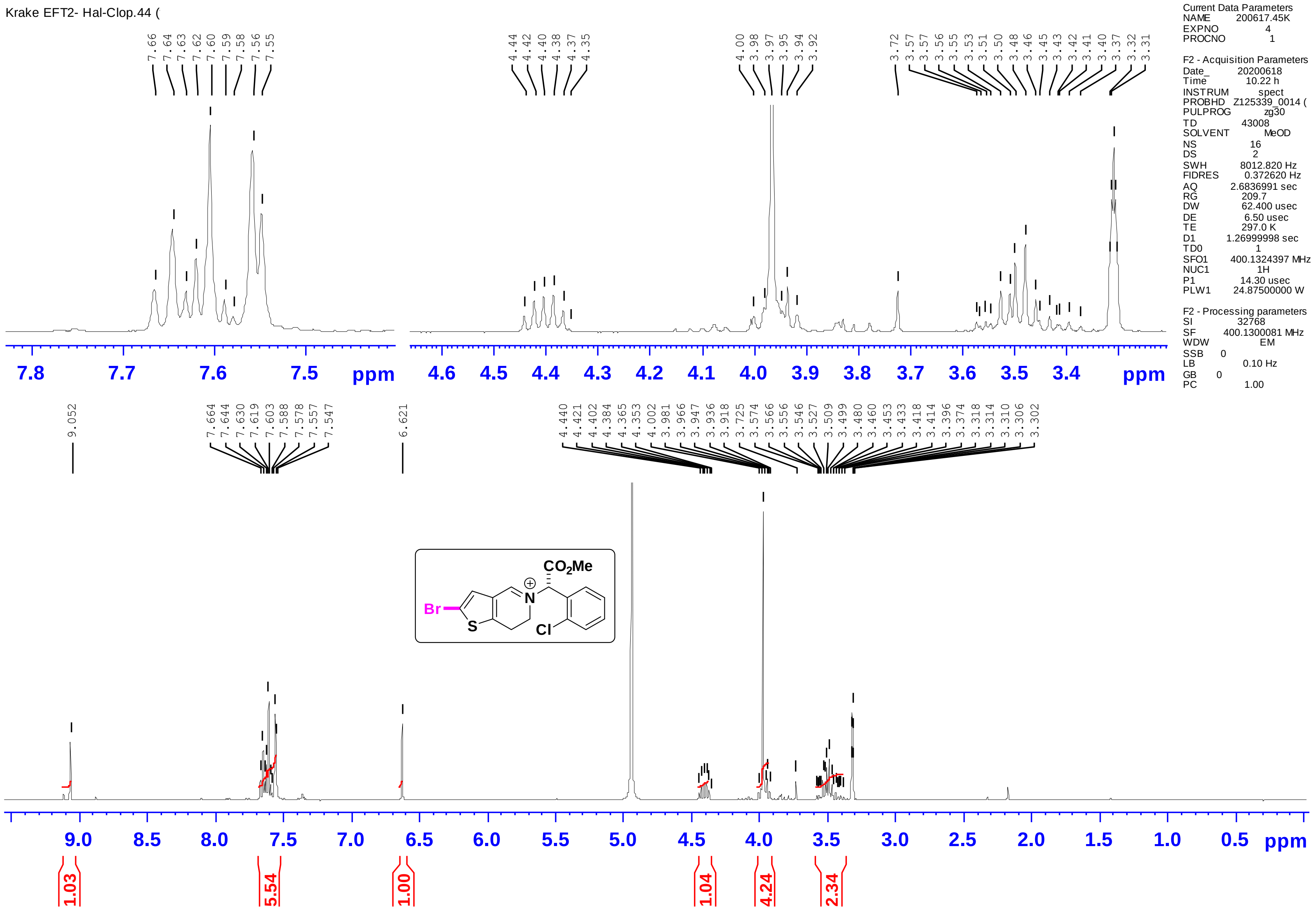

Figure S27. Copy of ^{1}H NMR spectrum of compound **DP-5b**.

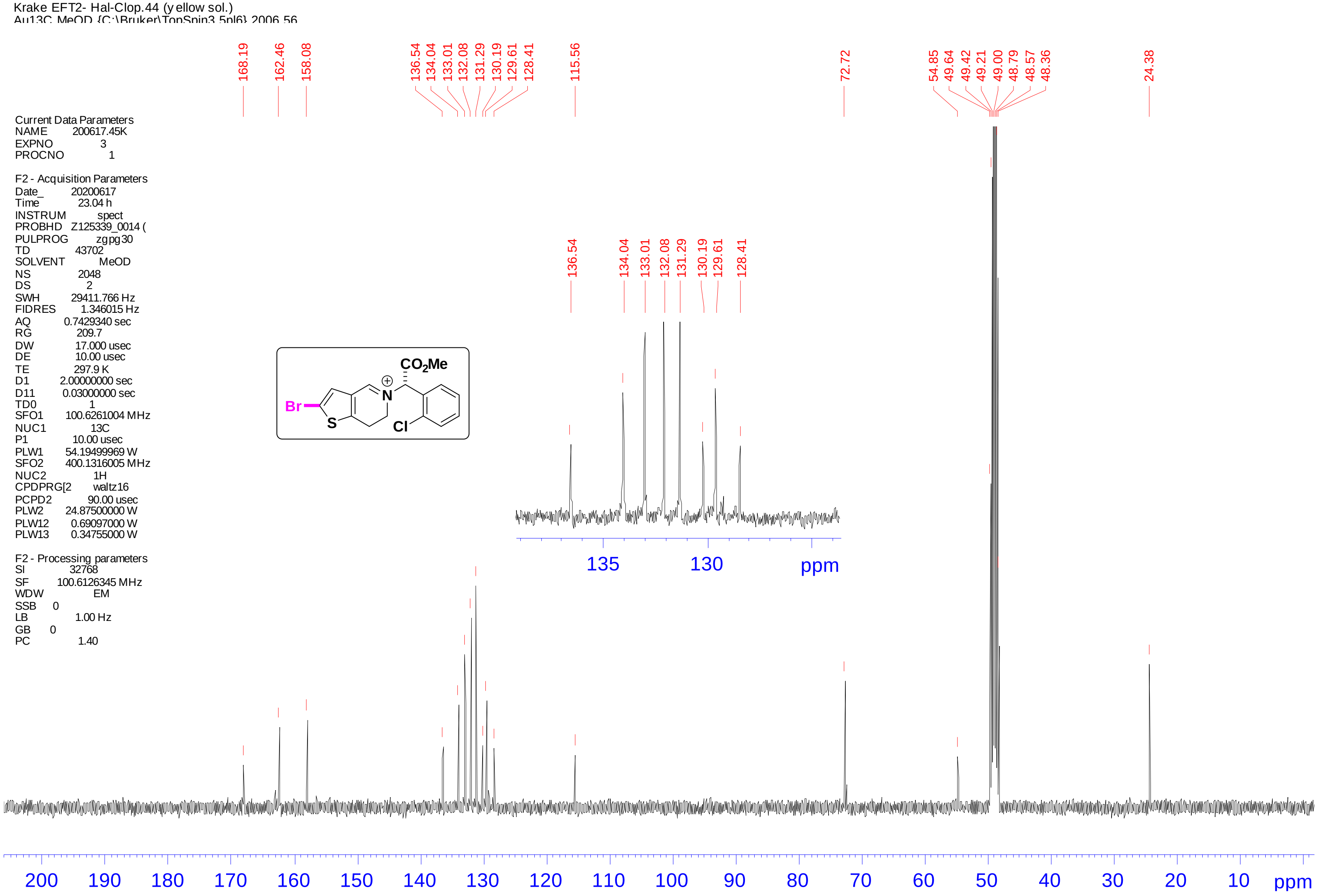

Figure S28. Copy of ^{13}C NMR spectrum of compound **DP-5b**.

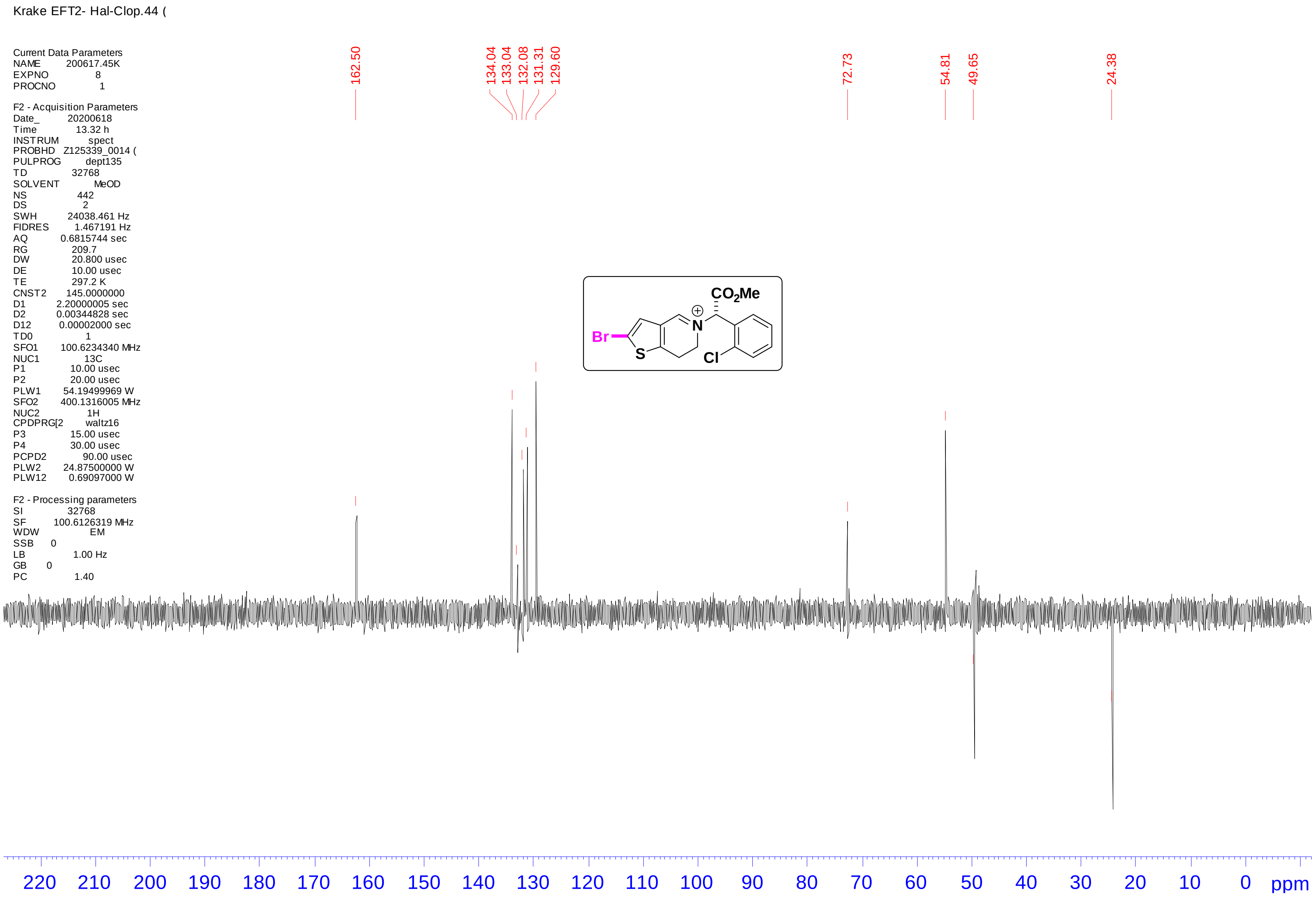

Figure S29. Copy of DEPT 135 spectrum of compound **DP-5b**.

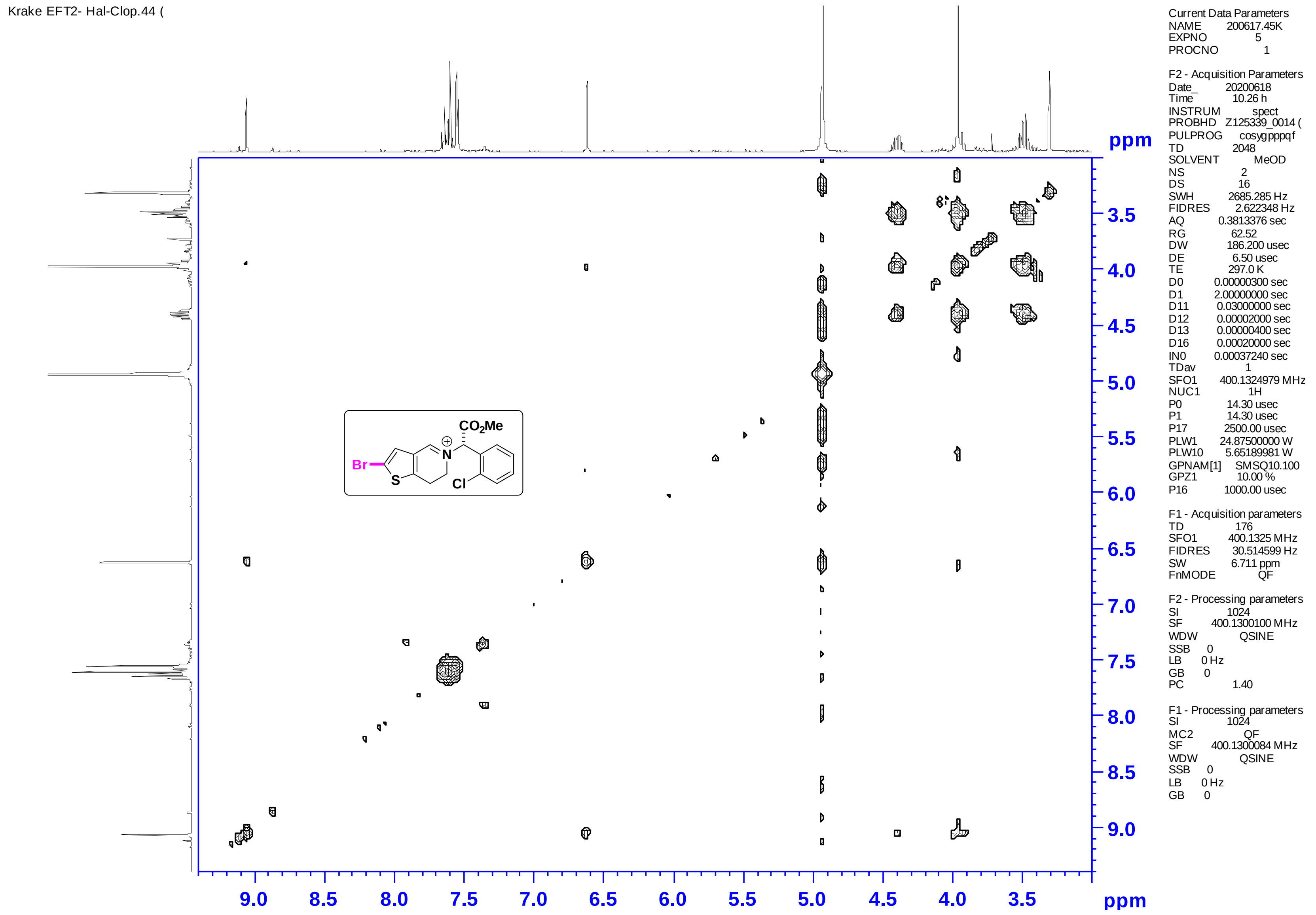

Figure S30. Copy of $^1H/^1H$ COSY spectrum of compound **DP-5b.**

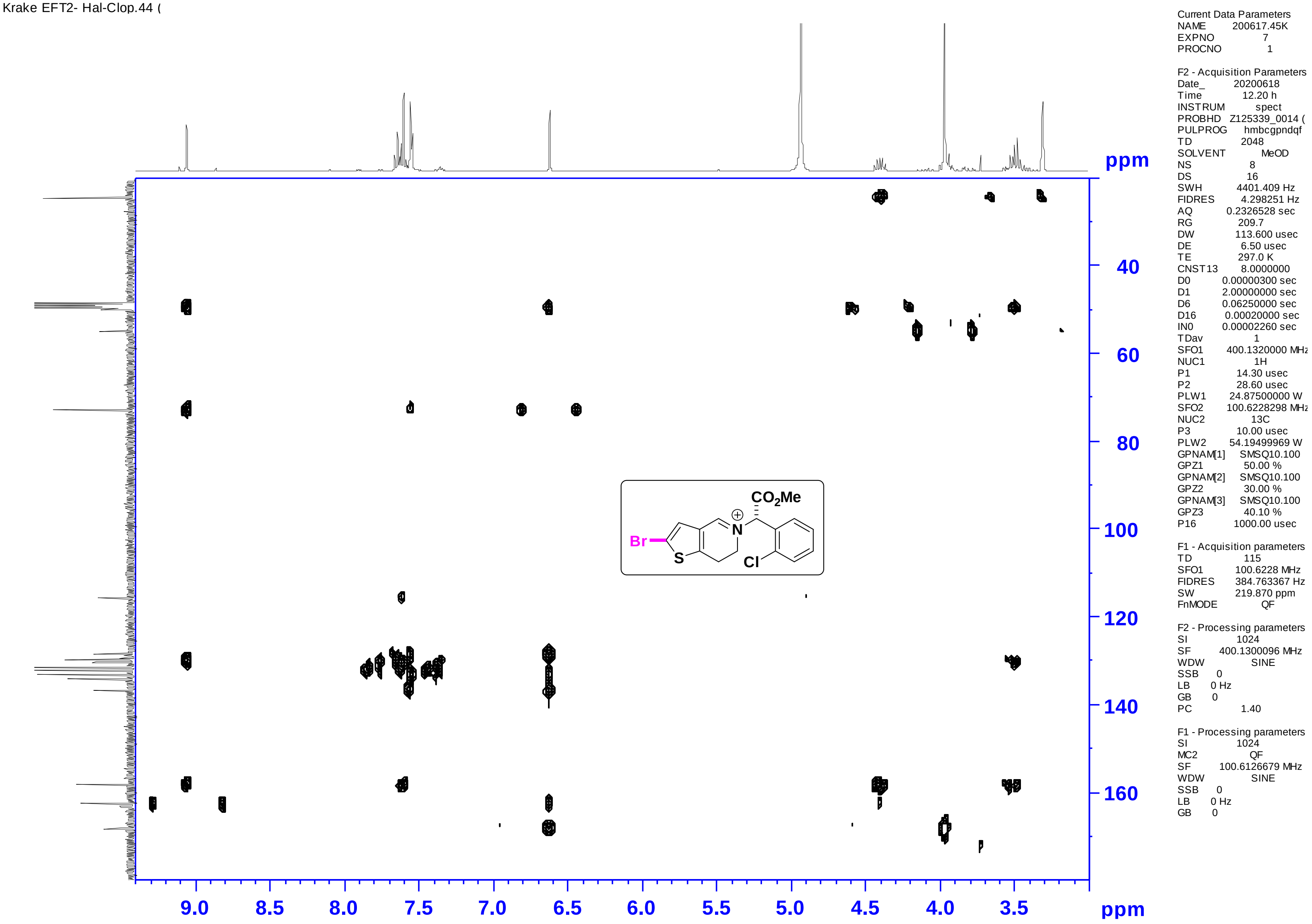

Figure S31. Copy of $^{1}H/^{13}C$ HMBC spectrum of compound **DP-5b**.

Sample Report:

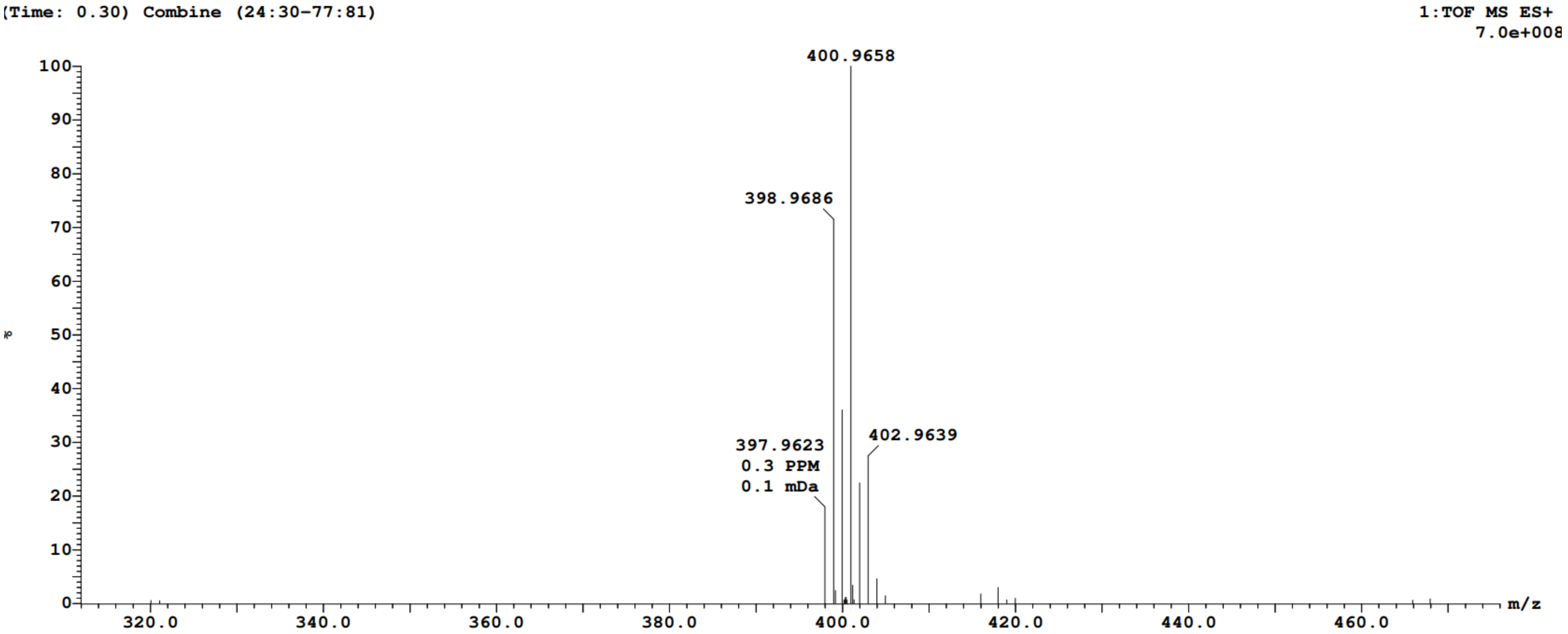

Figure S32. Copy of HRMS spectrum of compound **DP-5b** ($C_{16}H_{14}BrClNO_2S^+$, calc.: 397,9622, obs.: 397,9623; 0.3 ppm error).
